# Genome-Wide Association Study Reveals Genomic Regions Associated With Ten Agronomical Traits in Wheat Under Late-Sown Conditions

**DOI:** 10.3389/fpls.2020.549743

**Published:** 2020-09-17

**Authors:** Sundeep Kumar, Jyoti Kumari, Nabin Bhusal, Anjan Kumar Pradhan, Neeraj Budhlakoti, Dwijesh Chandra Mishra, Divya Chauhan, Suneel Kumar, Amit Kumar Singh, Mathew Reynolds, Gyanendra Pratap Singh, Kuldeep Singh, Sindhu Sareen

**Affiliations:** ^1^ ICAR-National Bureau of Plant Genetic Resources, New Delhi, India; ^2^ ICAR-Indian Institute of Wheat and Barley Research, Karnal, India; ^3^ ICAR-Indian Agricultural Statistics Research Institute, New Delhi, India; ^4^ International Maize and Wheat Improvement Center (CIMMYT), El Batan, Mexico

**Keywords:** terminal heat tolerance, association mapping panel, GWAS, SNPs, marker-assisted selection

## Abstract

Poor understanding of the genetic and molecular basis of heat tolerance component traits is a major bottleneck in designing heat tolerant wheat cultivars. The impact of terminal heat stress is generally reported in the case of late sown wheat. In this study, our aim was to identify genomic regions for various agronomic traits under late sown conditions by using genome-wide association approach. An association mapping panel of 205 wheat accessions was evaluated under late sown conditions at three different locations in India. Genotyping of the association panel revealed 15,886 SNPs, out of which 11,911 SNPs with exact physical locations on the wheat reference genome were used in association analysis. A total of 69 QTLs (10 significantly associated and 59 suggestive) were identified for ten different traits including productive tiller number (17), grain yield (14), plant height (12), grain filling rate (6), grain filling duration (5), days to physiological maturity (4), grain number (3), thousand grain weight (3), harvest index (3), and biomass (2). Out of these associated QTLs, 17 were novel for traits, namely PTL (3), GY (2), GFR (6), HI (3) and GNM (3). Moreover, five consistent QTLs across environments were identified for GY (4) and TGW (1). Also, 11 multi-trait SNPs and three hot spot regions on Chr1Ds, Chr2BS, Chr2DS harboring many QTLs for many traits were identified. In addition, identification of heat tolerant germplasm lines based on favorable alleles HD2888, IC611071, IC611273, IC75240, IC321906, IC416188, and J31-170 would facilitate their targeted introgression into popular wheat cultivars. The significantly associated QTLs identified in the present study can be further validated to identify robust markers for utilization in marker-assisted selection (MAS) for development of heat tolerant wheat cultivars.

## Introduction

Wheat is an important staple food crop supplying about 20% of the calories needed by the world population (Lobell and Gourdji, 2012; Shiferaw et al., 2013). In the wake of global warming, heat stress has emerged as one of the major constraints in wheat production around the world. Heat stress refers to plants exposed to high temperature during the grain filling period. The flowering under normal sown wheat (first fortnight of November North West plain zone) starts somewhere during mid-February and matures by mid-April. The average maximum and minimum temperatures during this period remains 25–30°C and 13–14°C although the maximum temperature touches 37/38°C and minimum temperature 20–23°C for few days towards the end of the grain filling period. However, under late sowing (during January) the average maximum temperature is 31–34°C with maximum limit above 40°C (40–43°C), and the average minimum temperature is 15–17°C with maximum limit of 23–24°C. January sown crop is exposed to high temperatures within one week of flowering and experiences stress for the most period of grain filling. Yield losses are caused by long periods of high temperatures as well as short heat waves during heading and grain filling stages (Wardlaw and Wrigley, 1994). Heat stress causes premature loss of photosynthetic capability due to accelerated senescence of leaves, over-dependence on stored carbohydrates (soluble sugars) in the stem in order to maintain grain filling, and lowers the plant’s ability to convert sugars to grain biomass (starch) due to heat-sensitivity of soluble starch synthase in the developing grain, which in turn severely affects yield and associated parameters (Jenner, 1994). The reproductive stages of wheat (flowering and grain filling) are highly sensitive to heat stress, and rises in temperature during this period can result in a complete loss of grain production (Zinn et al., 2010). As a result of high temperature, the grain filling duration is shortened, which affects the number of fertile spikelet’s, grain filling, and finally the yield (Dias and Lidon, 2009; Garg et al., 2013). Furthermore, heat stress also adversely affects pollen viability and fertilization. Average wheat yield losses in India due to a 1°C increase in temperature have been estimated at 9.1 ± 5.4% (Zhao et al., 2017), while global yield reduction caused by the same 1°C increase is estimated at 5.5%, signifying a total loss of 35 M tons (Lobell and Gourdji, 2012).

Enhanced tolerance to heat stress and increase in yield potential should remain priorities for the genetic improvement of wheat in response to climate change. Recent studies of grain set under high temperature indicate that considerable genetic variability exists, with little apparent yield penalty associated with improved heat tolerance (Sharma et al., 2017). To broaden the genetic base of existing wheat varieties, there is a need to characterize the germplasm lines for tolerance to terminal heat stress. In fact, the large wheat germplasm collection maintained by the national and international institutions is a rich repertoire of novel genes for economically important traits including tolerance for abiotic stresses such as heat, drought, and salinity. Evaluation of these collections may reveal novel genes/alleles conferring abiotic stress tolerance. In this regard, ICAR-National Bureau of Plant Genetic Resources (ICAR-NBPGR), New Delhi, which houses India’s National Genebank, has already initiated evaluation of about 20,000 wheat germplasm lines conserved for various traits to facilitate their utilization in breeding programs (Dutta et al., 2015; Kumar et al., 2016).

In general, the QTL mapping approach has been very successful with the biotic stresses particularly rust, powdery mildew, and spot blotch. Moreover, genes/QTLs for agronomical traits such as tiller number and thousand-grain weight have also been identified. In contrast, there has been limited success with abiotic stresses such as, drought and heat stress due to their complex genetic regulation. Furthermore, biparental QTL mapping generates low-resolution maps, and identified markers are not tightly linked to traits and therefore often fail to work reliably in MAS programs. However, Bi-parental QTL mapping gives high resolution maps in case of large progeny when used on high density platform. As a result, current gene mapping efforts are shifting from conventional biparental based mapping to linkage disequilibrium (LD) based association mapping that is genome-wide association (GWA) mapping (Zhu et al., 2008; Huang and Han, 2014). Association mapping exploits the ancestral recombination events at the population level (Yu and Buckler, 2006) and thus, provide an opportunity to identify potential candidate genes from the associated genomic regions. In wheat, there are several reports of association mapping based molecular dissection of traits including agronomic and yield associated traits (Breseghello and Sorrells, 2006; Liu et al., 2010), *Stagonospora nodorum* blotch resistance (Tommasini et al., 2007), Russian wheat aphid (Peng et al., 2009), grain yield under water stressed conditions (Tommasini et al., 2007), seedling and adult plant resistance to stripe rust (Zegeye et al., 2014; Gao et al., 2016; Muleta et al., 2017), spot blotch resistance in hard winter wheat (Ayana et al., 2018) and stripe rust resistance in emmer wheat (Liu et al., 2017). In recent years, there has been greater emphasis on deciphering the genetic and molecular basis of heat tolerance associated traits. Various studies have identified QTLs for heat tolerance component traits in wheat using association mapping panels (Paliwal et al., 2012; Bhusal et al., 2017; Qaseem et al., 2018). However, since heat tolerance is a highly complex trait, QTLs identified under a particular environmental condition might not be useful at other location/environments. Hence, there is a need to identify heat tolerant genomic regions/QTLs of wheat for the major wheat growing environments of the world and to accelerate the breeding of heat tolerant varieties for these regions.

Keeping the above in mind, the present study was conducted with the aim to identify genomic regions/QTLs associated with agronomical traits under late sown conditions using a diverse panel of wheat genotypes, mainly Indian wheat germplasm lines. The genomic regions identified in this study could be useful targets for the development of heat tolerant lines by wheat breeders.

## Materials and Methods

### Experimental Material

A wheat association mapping (AM) panel of 205 diverse genotypes including Indian landraces, indigenous and exotic germplasms, advance breeding lines, and some Indian wheat varieties released after 1960 (**Table S1**) was used for this analysis. The landraces, indigenous, and exotic germplasm lines were collected from the National Genebank of India located at ICAR-NBPGR, New Delhi. The advanced wheat breeding lines (RAJ3765/P11632; HD2808/HUW510) used in this analysis were developed at ICAR-Indian Institute of Wheat and Barley Research (IIWBR) Karnal.

### Experimental Design and Trait Evaluation

The wheat AM panel was evaluated at three different locations in the Indo-Gangetic plain region of India *viz*., ICAR-IIWBR, Karnal (29.43°N, 76.48°E, 245 msl), ICAR-IIWBR regional station Hisar (29.18°N, 75.70°E, 212 msl) and ICAR-NBPGR, New Delhi (28.24°N, 76.50°E, 190.7 msl). Field trials were carried out for three consecutive years from 2015 to 2017 during winter season (popularly known as *Rabi* season in India). Planting was done in three replications at each location. The planting was done from January 5th to 15th (normal date of sowing 15th November) with 5 days interval between the sowing of early, medium, and late maturing germplasm so that all the genotypes with different phenology included in AM could be exposed to heat stress at same growth phase. The experiment was laid out in Augmented Block Design with five checks (WH1124, HD3059, WH1105, PBW590, and DBW71) repeated in each block consisting of 46 germplasm lines. Each accession was sown in standard plot size that is 3.00 m^2^ under irrigated conditions. Recommended fertilizer doses (120 kg N: 60 kg P2O5: 40 kg K2O in per hectare area) were applied in field. The daily maximum and minimum mean temperatures were recorded for the entire crop season. Mean of minimum and maximum temperatures before and after heading was calculated by taking into consideration for each line, the minimum number of days to heading and maximum number of days to maturity. Propicanozole, a fungicide was applied to protect the crop from fungal disease.

### Phenotyping

Data were recorded for phenological, physiological, grain yield, and its component traits such as days to heading (DH), days to anthesis (DA), days to physiological maturity (DM), chlorophyll fluorescence (CFL), cell membrane stability (CMS), grain filling duration (GFD), grain weight/spike (GW, g), grain numbers/spike (GN), grain numbers/m (GNM), productive tillers/m (PTL), plant height (PHT, cm), 1,000 grain weight (TGW, g), biomass (BM, gm^−2^), grain yield (GY, gm^−2^), grain filling rate (GFR), and harvest index (HI, %) to find out the variation among studied wheat accessions.

The phenological traits were estimated at 50% growth stage on whole plot basis. GFD was estimated as difference in days between anthesis and physiological maturity. Five random main spikes were harvested from each plot followed by hand threshing to determine GN and GW estimation. TGW was measured by taking random samples of 1,000 grains/plot from plot yield and weighed. CFL was measured using fluorometer (Model OS 30P, Opti-Sciences, Hudson, NH, USA). The fluorescence data were recorded from three competitive plants tagged in the middle rows of the plot. The measurements were taken on the abaxial surface of fully expanded flag leaf at different growth stages (GS), flowering (GS65), end of flowering (GS70), medium milk (GS75), and early dough (GS83) as explained by Zadoks et al. (1974). Chlorophyll fluorescence values were recorded using clips, which created dark conditions for at least 20-min before measurements were taken. This is required for Q_A_, the primary stable electron acceptor of photosystem (PS) II reaction center, to be fully oxidized and enabled us to measure the minimal fluorescence (F_O_). Three readings per genotype per replication were recorded. The CMS was determined from three flag leaves of each genotype using methodology given in Sareen et al. (2019).

### Statistical Analyses

Analyses of variance and least square means of all traits measured over the different locations and years were estimated using the SAS PROCGLM (SAS software v9.3). Heritability of the analyzed traits was estimated using restricted maximum likelihood (REML) method. For each location, trait mean and ranges of each accession were calculated. BLUP values were calculated using R package for further statistical analysis. The phenotypic correlation coefficients (r^2^), scatter plot matrix and principal component analysis for all 16 morpho-physiological traits were analyzed using SAS software v9.3 program. The biplots based on two principal components were also generated to depict the accession scores as well as loading of characters. Genotype and genotype by environment (GGE) biplot analysis was performed to determine genotype stability, superiority and also ‘which-won-where’ plot for trait based best genotypes at a particular location in relation to important agronomical characters under heat stress using R program (Yan and Tinker, 2006).

### DNA Extraction and SNP Genotyping

The genomic DNA was extracted from 15 days old seedlings using a CTAB procedure (Doyle and Doyle, 1990). Genotyping of the association panel was performed using Axiom Wheat Breeders’ Array according to the procedure described by Affymetrix (Axiom 2. 0 Assay for 384 samples P/N 703154 Rev. 2). Allele calling was done using the Axiom Best practices genotyping workflow (http://media.affymetrix.com/support/downloads/manuals/axiom_genotyping_solution_analysis_guide.pdf). All the SNP markers with a call rate < 90% and minor allele frequency (MAF) < 10% across all genotypes were excluded from the analysis. To assign the physical location of each SNP on wheat chromosomes, the SNP probe sequences were BLAST searched against the wheat genome assembly *RefSeq* v1.0 (https://wheat-urgi.versailles.inra.fr/Seq-Repository/Assemblies).

### LD, Population Structure, and Kinship Analysis

LD between each pair of markers was estimated as the squared allele frequency correlation (*r2*) using software TASSEL v. 5.0 (Bradbury et al., 2007). LD decay distance across the subgenomes and whole genome was estimated by plotting the scatterplot of LD *r2* values between marker pairs and the physical distance. The background LD in wheat AM panel was calculated to identify critical distance for LD decay. Population structure was analyzed using a model-based clustering approach implemented in STRUCTUREv2.3.4 software (Pritchard et al., 2000). For each specified K, ten iterations of STRUCTURE were conducted with additional parameters 20,000 burn-in length and 50,000 Markov Chain Monte Carlo (MCMC) iterations. The optimum number of subpopulations (K) was estimated using *ad-hoc* statistics ΔK (Evanno et al., 2005). The kinship matrix (K), which is based on the scaled IBS (identity by state) method was also estimated using Tassel 5.0 (Bradbury et al., 2007).

### Genome-Wide Association Analysis

Marker trait associations (MTAs) were estimated using Mixed Linear Model (MLM) implemented in TASSEL 5.0 (http://www.maizegenetics.net/). The MLM approach was used as it takes into account population structure (Q) and kinship matrix (K) as covariates (MLM: Q + K) thus minimizing the chances of getting spurious associations. Initially, the significant associations were detected at threshold *P*-value < 0.0001. Further, raw *P*-values were adjusted with Bonferroni correction (P-value < 4.19E-06) to detect significantly associated QTLs with traits. In addition, LD among the clustered SNPs was estimated, and those with high LD were considered single QTLs. The R package qqman (https://cran.r-project.org/package=qqman) was used to draw Manhattan plots. The favorable alleles for each QTL region were identified by comparing the extreme phenotypic values in association mapping panel.

## Results

### Phenotyping of Wheat Association Panel

The analysis of variance (ANOVA) based on individual evaluation data of three years (2015, 2016, and 2017) and three locations revealed significant differences among the genotypes for all the studied traits (**Table 1**). Locations also showed significant differences for the analyzed traits whereas for the year, it was significant for DA, GW, TGW, HI, CFL, and GFR. Further, the majority of the analyzed traits followed normal distribution (**Figures S1A–P**) and also showed wide variations based on descriptive statistics (**Table S2**). The average GN ranged from 19.6 to 60.2 at Delhi, 22 to 80 at Hisar, and 20.2 to 64.8 at Karnal. The average PTL varied from 28 to 107, 56 to 88 and 39 to 123 at Delhi, Hisar, and Karnal respectively. For other traits the association panel showed wide variability as well.

**Table 1:** ANOVA of traits evaluated at three locations under late sown conditions.

SOURCE	DF	DH	DA	DM	GFD	PHT	PTL	GN	GNM
Location	2	590.534 ***	622.324 ***	303.840 ***	359.998 ***	1,934.435 ***	38,005.283 ***	11,529.321 ***	134,423,447.520 ***
Year	2	2.724 NS	79.804 ***	6.571 ***	23.494 ***	7.841 NS	5.404 NS	917.014 NS	888,717.141 NS
Treatment	204	260.32 ***	231.740 ***	389.660 ***	68.454 *	3997.176 ***	83,779.896 ***	1,286.757 ***	98,373,280.216 ***
Loc*Yr	4	3.166 NS	5.021 NS	11.249 NS	230.880 ***	148.816 *	11.686 NS	0.518 (0.07)	8,9828.662 (1.00)
Loc*Trt	408	188.137 ***	168.013 NS	75.740 ***	366.616 ***	571.368 ***	10,742.046 **	284.143 NS	26,038,106.072 *
Yr*Trt	408	0.290 NS	0.660 NS	0.929 NS	0.791 NS	0.560 NS	1.039 NS	0.049 *	62,376.970 NS
Loc*Yr*Trt	816	0.313 NS	0.831 NS	0.537 NS	1.632 NS	0.790 NS	0.764 NS	0.045 NS	12,243.926 NS
Error	1,226	13.050	8.698	10.314	21.670	51.803	4280.003	185.000	11,304,041.843
**SOURCE**	**DF**	**GW**	**TGW**	**BM**	**GY**	**HI**	**CFL**	**CMS**	**GFR**
Location	2	2.733 ***	234.220 ***	30,502,312.393 ***	257,958.638 ***	1,999.703 ***	0.029 ***	1,339.568 ***	18,673.249 ***
Year	2	5.468 ***	42.187 ***	1,1617.771 NS	661.960 NS	930.440 ***	0.053 ***	34.622 NS	1,535.681 ***
Treatment	204	2.525 ***	631.140 ***	1,943,725.900 ***	49,8474.712 ***	721.679 ***	0.021 ***	7,739.173 ***	278.607 ***
Loc*Yr	4	0.573 ***	113.812 ***	80,583.059 NS	3,253.960 NS	1,362.209 ***	0.163 ***	34.540 NS	3,026.402 ***
Loc*Trt	408	1.253 ***	167.458 ***	432,068.652 ***	247,582.702 ***	221.223 ***	0.047 ***	1,178.309 ***	80.579 NS
Yr*Trt	408	0.020 NS	6.230 NS	50.071 NS	38.093 NS	114.371 ***	0.002 ***	.199 NS	3.527 NS
Loc*Yr*Trt	816	0.010 NS	4.960 NS	54.173 NS	38.093 NS	111.510 ***	0.002 ***	.513 NS	2.994 NS
Error	1,226	0.056	7.725	47,852.000	12,376.630	18.164	0.004	22.667	57.090

NS, Non-Significant; DF, Degree of freedom.

*, ** and *** indicate 0.05, 0.01, and 0.001 levels of significance.

**Figure 1:**
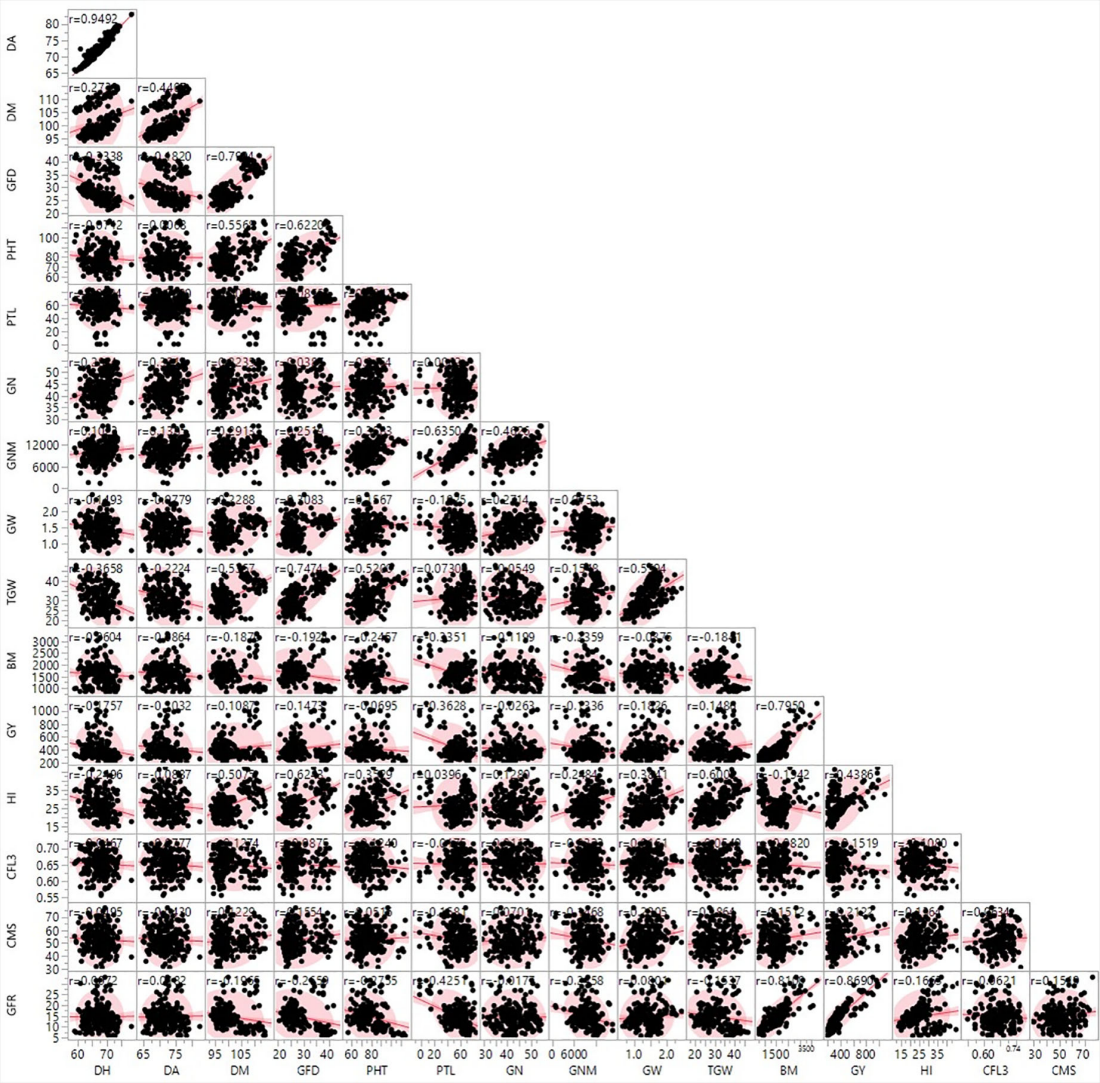
Scatter plot matrix showing correlation between 16 morpho-physiological traits of wheat genotypes under terminal heat stress condition. SAS software v9.3.

The correlation coefficient between all the studied traits was also analyzed (**Figure 1**). A highly significant positive correlation was observed between DH and DA (0.949), and DM and GFD (0.799). Some traits showed positive association with more than one trait such as GY, which was positively correlated with DM (0.108), GFD (0.147), GW (0.182), TGW (0.148),BM (0.795), HI (0.438), and GFR (0.869). TGW was also positively and significantly correlated with DM (0.535), GFD (0.747), GW (0.559), and HI (0.600).

PCA was performed to identify the measured parameter that best described the response under stress condition based on principal component score (**Figure S2**). PCA results showed that five factors had Eigen values > 1. Moreover 46.29% of the total variability was explained by the first two PCs under the extreme heat stress condition. The first PC was positively correlated with DM, GFD, PHT, PTL, GNM, GW, TGW, and HI, whereas PTL, GNM, and GFR contributed most to PC2. Wheat genotypes were classified into three groups based on biplots of PC1 *vs.* PC2 (**Figure S2a**). These genotypes were better differentiated when classified on the basis of TGW and GFD (**Figures S2B, C**). Therefore, under the extreme heat stress condition, genotypes, HD2888, IC611071, IC611273, IC75240, IC321906, IC416188, and J31-170 were considered tolerant genotypes showing high scores for PC1 and PC2 and classified into group 1 while genotypes IC28658, IC78856, 11-F1-3, HGP1-318, HGP1-448, IC296681, HGP1-315, HGP1-306, HGP1-208, HGP1-470, J31-2, 11-F1-2, J31-145, F1-5, and IC533761 were categorized as moderately tolerant genotypes. Group I genotypes had high GFD and medium to high TGW, whereas the majority of group II genotypes had mostly high TGW and high GFD. The remaining genotypes were classified as moderately sensitive.

### GGE Biplot Analysis of Grain Yield Components and Their Stability in Wheat Association Panel

Trait-wise GGE biplot analysis was performed for yield component traits namely HI, PTL, GFD, GN, GY, and TGW to identify the best genotypes and their stability across the three locations (**Figures S3A–C, S4A–C**). For PTL, IC279335 and J31-170 were the best performing genotypes at Hisar location, whereas J31-73 and IC112049 performed best in Delhi and Karnal. Genotypes IC290246, J31-23, IC547561 were specifically suitable for GFD at the Delhi location, whereas IC290196, IC335712, and IC60221 at Hisar and J31-73 at Karnal. For GN, IC75242 and IC252655 were identified as best performing genotypes at the Delhi location, whereas K8027 and IC28658 performed best at Hisar and IC290080 and IC534596 performed best at Karnal. Similarly, genotypes IC533903 and K8027 were best performing genotypes for GY at the Delhi location whereas IC533654 at Hisar, and IC445595 and HD2932 at Karnal. For HI, the best performing genotypes specifically suited to the Delhi location were IC128335 and IC128218, whereas IC290080 performed best at Hisar; IC335523 and IC539565 performed best at Karnal. Promising genotypes for TGW at the Delhi location were IC335690, IC539602, IC138852, IC398010, IC335742, whereas IC535176 was most promising genotype at Karnal. HGP1-32, IC60221, and IC416089 were promising genotypes at Hisar.

In mean *vs.* stability analysis, the highest mean value for PTL was recorded for IC36761 followed by IC611479 and IC252472, which were consistently more tolerant. On the other hand, IC531970, K8027, and IC145428 were consistently most susceptible. The most stable genotypes were IC539600 and IC335534, which had above average performance. Similarly, for GFD, stable genotypes with above average performance were C306 (check), IC539292, and IC128573. Stable genotypes for GN were EC190950 and IC536060, whereas for GY, IC82460, IC335690, and IC539600 showed stable and above average performance. For TGW and HI the stable genotypes were IC36761A, HGP1-107, and IC547662, IC543417, IC112049 and 11-F1-8, respectively.

Ranking of genotypes on the basis of GGE biplot may be determined as distances from ideal genotype. For PTL, IC93335 was the most favorable genotype followed by IC539599 (**Figures S3A–C, S4A–C**). Similarly, other genotypes may be ranked in decreasing order by observing distances from the center of the circle. IC542901 was most favorable genotype for GFD followed by IC111905, IC335677, and IC539600, whereas genotype HGP1-32 was most suitable for GN. Similarly, for GY, the most suitable genotype was IC598225 followed by IC252897, IC445595, IC543356, and IC539565. For TGW, the highest ranked genotypes were in the order of IC28665, HD2932, IC290173, 11-F1-3, HGP1-208, and IC416141. For HI, J31-2 was the most suitable genotype.

### Marker Coverage, Population Structure, and Linkage Disequilibrium

Genotyping of the AM panel using 35 K Axiom SNP array revealed a total of 23,053 scorable SNPs after filtering for various quality parameters. SNPs with MAF ≤ 0.1 were then excluded, which left 15,886 high quality SNPs for further analyses. These SNPs (probe sequences) were searched against the reference wheat genome assembly, IWGSC (International wheat genome sequencing consortium) *RefSeq* v1.0 (https://wheat-urgi.versailles.inra.fr/Seq-Repository/Assemblies) using BLASTN program with default parameters. A total of 11,911 SNPs was assigned exact physical location on wheat genome which included 3,729 on subgenome A, 4,760 on subgenome B, and 3,422 on subgenome D. The number of SNP markers on individual chromosomes ranged from 210 on Chr4D to 883 on chromosome Chr2B. The chromosomal level distribution of 11,911 SNPs on three subgenomes showed that subgenome A possessed maximum SNPs on Chr2A (780) followed by Chr1A (708) and Chr7A (618). Subgenome B contained maximum SNPs on Chr2B (883) followed by Chr1B (842) and Chr5B (807), whereas subgenome D contained maximum markers on Chr2D (712) followed by Chr1D (673), and Chr5D (524). The SNPs that could be assigned exact physical locations on the wheat reference genome were only considered for the further analyses in order to exclude any conflict with homoeologous SNPs that might be present across the three wheat subgenomes.

To investigate pair-wise linkage among markers, chromosome-wise LD plot was generated for 11,911 SNP markers selected for association analysis (**Figures S5A–D**). The LD (r^2^) across 21 wheat chromosomes ranged from 0.078 (Chr4D) to 0.349 (Chr1B). In contrast, the subgenome level LD estimate did not differ much: 0.15 for subgenome A, 0.17 for subgenome B, and 0.16 for subgenome D. The summary statistics of 11,911 markers including chromosome wise distribution, average LD, average marker distance, and markers with perfect LD are presented in **Table S3**. These results indicated the presence of varying levels of LD across different chromosomes within each subgenome. The decay of LD across the genome is an important parameter that determines the number of significant markers required for performing GWAS analysis. The background LD in the analyzed AM panel was equal to 0.167 which was taken as threshold cut-off for estimating LD decay. The whole genome level LD in AM panel decayed at 6.4 Mb. Further, LD decayed the fastest in subgenome A followed by subgenomes B and D. In the case of subgenome, A, r^2^ value for the marker pairs reached 0.167 at 3.2 Mb as compared to 4.3 Mb in subgenome B and 6.8 Mb in subgenome D (**Figures S5A–D**).

**Figure 2:**
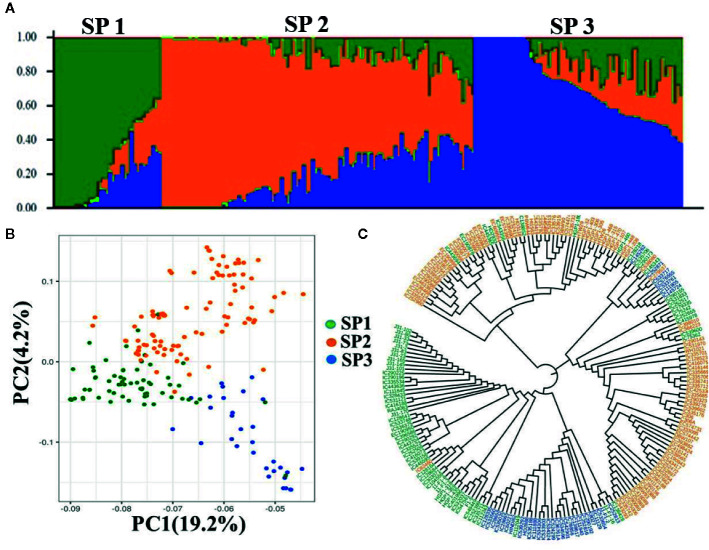
Population structure, PCA, and NJ tree of wheat association mapping panel **(A)**. Population Structure. Bar graphs for three subpopulations are indicated by different colors. The vertical coordinates of each subpopulation indicate the membership coefficient for each individual, and the digits on the horizontal coordinates represent the corresponding genotypes corresponding to the table. In each subpopulation, each vertical bar represents one genotype. STRUCTUREv2.3.4 (https://web.stanford.edu/group/pritchardlab/structure_software/release_versions/v2.3.4/html/structure.html). **(B)** PCA ggplot2 R program (https://cran.r-project.org/web/packages/ggplot2/citation.html) and **(C)** NJ tree.

Population structure indicates the level of genetic differentiation among a group of individuals/genotypes. Population structure analysis of the selected wheat AM panel using all the 15,886 SNPs revealed three subpopulations (K = 3) containing 98, 68, and 39 genotypes, respectively (**Figure 2A**). Individuals of each subpopulation were further categorized as pure and admixture types. Genotypes that had a membership proportion of ≥0. 8 were considered pure, and genotypes <0.8 were considered admixtures. Based on this criterion, the composition of three subpopulations was as follows; subpopulation-1 (26% pure and 74% admixtures); subpopulation-II (30% pure and 70% admixtures), and subpopulation-III (28% pure and 72% admixtures). Interestingly, the three clusters were also present in the N-J based trees (**Figure 2B**), indicating consistency in the grouping of genotypes. The principal component analysis (PCA) of AM panel genotypes was performed to estimate population structure including the first three PCs. PC1 explained 19.2% of the genetic variance whereas PC2 explained 4.2%. The PC scatter plot (**Figure 2C**) showed that the first and second PCs were composed largely of three subpopulations of genotypes originating from different regions.

### Genome-Wide Association Analysis

**Figure 3:**
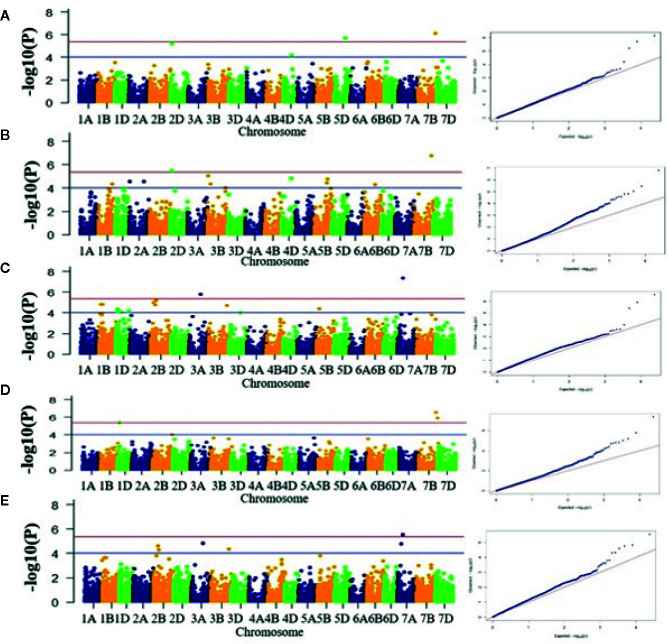
Manhattan plots for five different traits. **(A)** DM, **(B)** PHT, **(C)** HI, **(D)** GY, and **(E)** GFR. X-axis shows position of SNP markers on 21 chromosomes (1A to 7D) and Y-axis shows significance of the association tests on a −log scale. Blue horizontal line represents the draw P value threshold (P value < 01E-4). Red dash line indicates Bonferroni corrected *P* value (*P* value < 4.19E-6). Manhattan plots were generated in the qqman R package, v0.1.4 (https://cran.r-project.org/web/packages/qqman/index.html).

**Figure 4:**
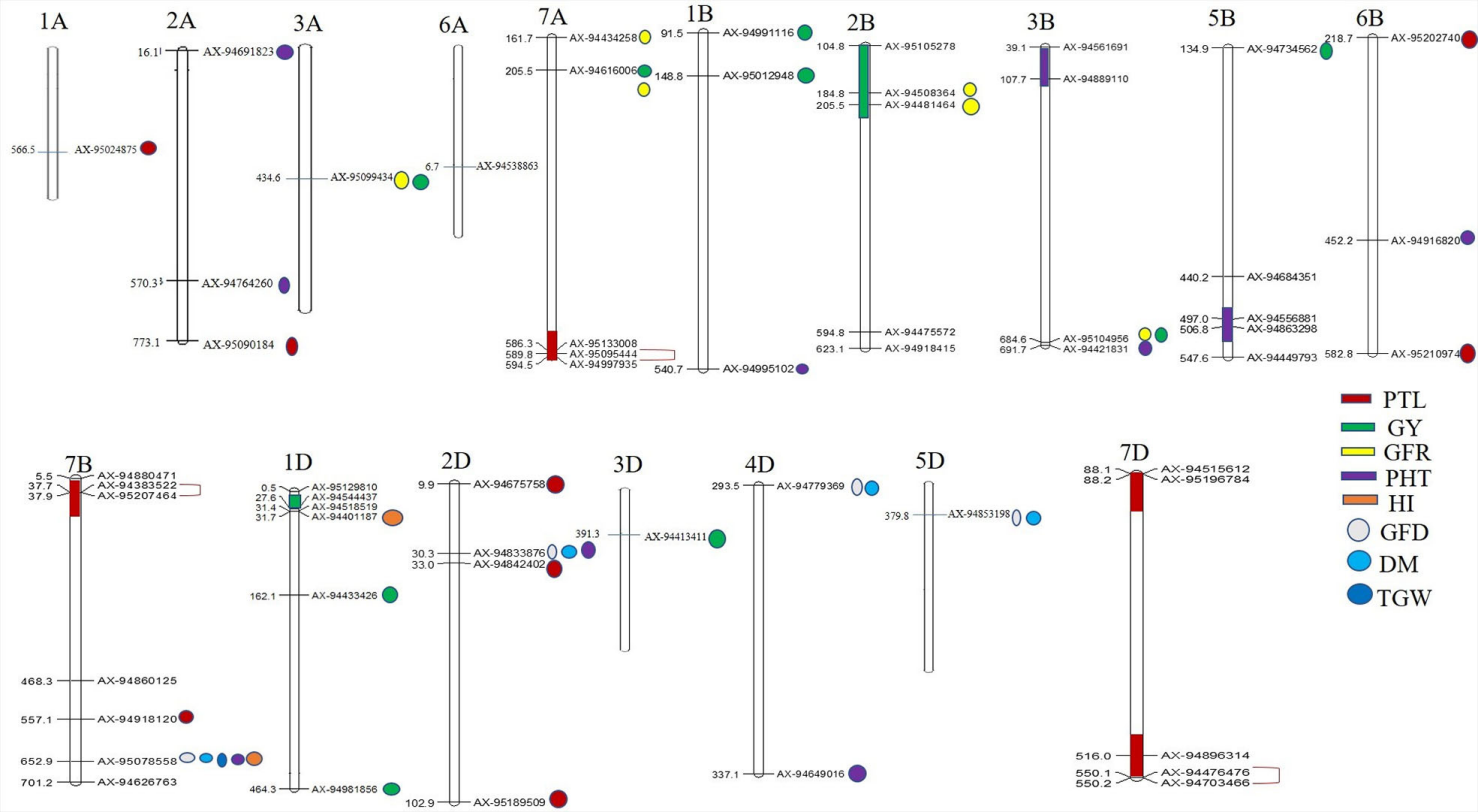
Physical locations of SNPs significantly associated with 10 different traits. The regions possessing two or more MTAs for a trait have been highlighted with color. SNPs in complete LD are shown with red color single bracket symbol. Mapchart v2.23 (https://www.wur.nl/en/show/Mapchart.htm).

A total of 69 associations were detected for 10 out of 16 analyzed traits under late sown conditions at P-value significant threshold (<0.0001) (**Figures 3A–E**, **Figures S6A–E**, **Table 2**). However, out of these 69 associations, only 10 associations were significant after applying Bonferroni correction (P-value < 4.19E-6). We considered these 10 associations as significantly associated QTLs for the respective traits, whereas the remaining 59 associations were designated as suggestive QTLs. Fit of the GWAS model was tested using quantile-quantile-plots (Q-Q plots) between observed and expected P-values of association that revealed a good fitting for the model with population structure and kinship (**Figures S7A–J**). The 69 identified QTLs were distributed on 17 wheat chromosomes representing three subgenomes *i.e.*, subgenome A (Chr1A, Chr2A, Chr3A, Chr6A, and 7A); subgenome B (Chr1B, Chr2B, Chr3B, Chr5B, Chr6B, and Chr7B); subgenome D (Chr1D, Chr2D, Chr3D, Chr4D, Chr5D, and Chr7D) (**Table 2**, **Figure 4**). The maximum number of QTLs was found on subgenome B (35) followed by subgenomes D (22) and A (12). Chromosome wise, the highest number of QTLs was detected on Chr7B (11) followed by Chr2B (7). Trait-wise distribution of 69 identified QTLs showed large variation ranging from two (BM) to 17 (PTL). The number of QTLs for other traits was: GY-14, PHT-12 GFR-6, GFD-5, DM-4, GNM-3, TGW-3 and HI-3 (**Table 2**). The most significant QTL was recorded for GY (AX-94616006, P-value = 4.05E-08) followed by PHT (AX-95078558, P-value =1.59E-07), and HI (AX-95078558, P-value = 2.75E-07).

**Table 2:** Details of 69 QTLs identified for 10 different traits under late sown conditions.

Sl.no	Trait	Marker	alleles	Chr	Pos (mbp)	P-value	R^2^ (%)
1	GFD	AX-94833876	G/A	2DS	30.252	7.50E-06	3.35
2	GFD	AX-94779369	C/A	4DL	293.546	8.61E-05	2.52
3	GFD	AX-94853198	A/C	5DL	379.79	7.32E-05	2.55
4	GFD	AX-94538863	C/T	6AS	6.735	6.18E-05	2.59
5	GFD	AX-95078558	C/T	7BL	652.934	5.28E-06	3.66
6	DM	AX-94833876	G/A	2DS	30.252	6.23E-06	3.42
7	DM	AX-94779369	C/A	4DL	293.546	6.45E-05	2.62
8	DM	AX-94853198	A/C	5DL	379.79	1.99E-06^*^	3.55
9	DM	AX-95078558	C/T	7BL	652.934	7.02E-07^*^	4.32
10	BM	AX-94475572	A/G	2BL	594.848	7.01E-05	2.54
11	BM	AX-94860125	C/G	7BL	468.294	9.88E-06	3.31
12	TGW	AX-94918415	A/G	2BL	623.056	2.62E-05	3.62
13	TGW	AX-94684351	C/T	5BL	440.238	1.15E-05	3.49
14	TGW	AX-95078558	C/T	7BL	652.934	9.90E-06	3.67
15	PTL	AX-95072103	G/T	1AS	28.620	1.55E-04	2.14
16	PTL	AX-95090184	A/G	2AL	773.186	7.45E-04	1.86
17	PTL	AX-94675758	G/A	2DS	9.902	6.52E-04	1.44
18	PTL	AX-94842402	A/C	2DS	32.971	7.08E-04	1.71
19	PTL	AX-95189509	G/A	2DS	102.859	1.52E-04	2.17
20	PTL	AX-94449793	G/A	5BL	547.584	4.58E-04	2.05
21	PTL	AX-95210974	T/G	6BL	582.825	8.57E-04	1.73
22	PTL	AX-95202740	C/T	6BS	218.655	8.53E-04	1.73
23	PTL	AX-95133008	G/A	7AL	586.276	3.87E-04	1.85
24^#^	PTL	AX-95095444	G/T	7AL	589.756	3.35E-04	1.90
	PTL	AX-94997935	G/A	7AL	594.459	8.64E-04	1.70
25	PTL	AX-94918120	G/T	7BL	557.061	9.01E-04	1.75
26	PTL	AX-94880471	C/G	7BS	5.515	8.78E-04	1.94
27^#^	PTL	AX-94383522	C/T	7BS	37.855	4.34E-04	1.85
	PTL	AX-95207464	G/C	7BS	37.857	1.69E-04	2.09
28	PTL	AX-94896314	C/G	7DL	516.030	1.14E-04	1.76
29^#^	PTL	AX-94476476	A/G	7DL	550.098	4.28E-04	1.84
	PTL	AX-94703466	T/C	7DL	550.102	5.93E-04	1.75
30	PTL	AX-94515612	C/T	7DS	88.149	9.61E-05	1.83
31	PTL	AX-95196784	T/C	7DS	88.194	5.45E-05	1.94
32	PHT	AX-94995102	C/T	1BL	540.729	4.52E-05	2.44
33	PHT	AX-94691823	A/C	2AS	16.066	2.79E-05	2.54
34	PHT	AX-94764260	C/T	2AL	570.268	2.80E-05	2.54
35	PHT	AX-94833876	G/A	2DS	30.252	3.39E-06^*^	3.21
36	PHT	AX-94561691	A/C	3B	39.141	9.43E-06	3.02
37	PHT	AX-94889110	G/A	3B	107.666	4.64E-05	2.65
38	PHT	AX-94421831	T/A	3B	691.661	9.30E-05	2.28
39	PHT	AX-94649016	A/C	4DL	337.074	1.56E-05	2.77
40	PHT	AX-94556881	A/G	5BL	496.98	3.34E-05	2.54
41	PHT	AX-94863298	C/A	5BL	506.761	1.63E-05	2.7
42	PHT	AX-94916820	C/G	6BL	452.188	5.05E-05	2.41
43	PHT	AX-95078558	C/T	7BL	652.934	1.59E-07^*^	4.06
44	HI	AX-94401187	G/C	1DS	31.692	4.17E-06^*^	3.97
45	HI	AX-95078558	C/T	7BL	652.934	2.75E-07^*^	4.99
46	HI	AX-94626763	T/G	7BL	701.218	1.25E-06^*^	4.81
47	GY	AX-94991116	G/A	1BS	91.558	1.46E-05	2.73
48	GY	AX-95012948	T/G	1BS	148.898	1.46E-05	2.73
49	GY	AX-94544437	C/G	1DS	27.556	4.58E-05	2.45
50	GY	AX-94518519	G/A	1DS	31.431	5.61E-05	2.59
51	GY	AX-94433426	A/C	1DS	162.062	8.73E-05	2.23
52	GY	AX-94981856	A/G	1DL	464.319	6.35E-05	2.37
53	GY	AX-95105278	T/C	2BS	104.833	9.69E-06	2.8
54	GY	AX-94508364	G/A	2BS	184.8	1.69E-05	2.7
55	GY	AX-94481464	A/C	2BS	205.477	6.37E-06	2.88
56	GY	AX-95099434	A/G	3AL	434.615	1.61E-06^*^	3.3
57	GY	AX-95104956	T/G	3B	684.618	1.97E-05	2.74
58	GY	AX-94413411	G/T	3DL	391.263	9.35E-05	1.82
59	GY	AX-94734562	G/A	5BS	134.925	4.06E-05	2.46
60	GY	AX-94616006	C/A	7AS	205.485	4.05E-08^*^	4.32
61	GNM	AX-95129810	C/G	1DS	0.513	6.77E-05	2.84
62	GNM	AX-94449793	G/A	5BL	547.584	1.91E-05	3.5
63	GNM	AX-95207464	G/C	7BS	37.855	9.63E-05	2.82
64	GFR	AX-94508364	G/A	2BS	184.8	2.48E-05	2.24
65	GFR	AX-94481464	A/C	2BS	205.477	4.79E-05	2.06
66	GFR	AX-95099434	A/G	3AL	434.615	1.48E-05	2.37
67	GFR	AX-95104956	T/G	3B	684.618	4.71E-05	2.18
68	GFR	AX-94434258	T/C	7AS	161.738	1.74E-05	2.48
69	GFR	AX-94616006	C/A	7AS	205.485	2.84E-06^*^	2.77

*Indicates Bonferroni corrected P value (P valve < 4.19E-6), ^#^SNPs are in complete LD.

Ten significantly associated QTLs were represented by five important traits; three QTLs for HI (Chr7BL: AX-95078558, Chr7BL: AX-94626763, and Chr1DS: AX-944401187), two QTLs each for GY (Chr3AL: AX-95099434 and Chr7AS: AX-94616006), PHT (Chr7BL: AX-95078558, and Chr2DS: 94833876), DM (Chr5DL: AX-94853198 and Chr7BL: AX-95078558) and one QTL for GFR (**Figures 3A–E**, **Table 2**).

Furthermore, in order to identify consistent MTAs across environments, GWAS was separately performed for the three evaluated locations. The results are presented in **Supplementary Table S4**. A total of five QTLs were consistently present at Delhi and Karnal. These include four QTLs for GY (Chr6B: AX-94437800, Chr7A: AX-94616006, Chr2B: AX-94508364, and Chr3B: AX-95104956) and one QTL for TGW (Chr5A: AX-94955512).

### Co-Localized QTLs for More Than One Trait

Association of the same genomic region with multiple traits is a very common phenomenon in plants. We observed that a total of 12 SNPs was associated with more than one agronomical trait (**Table 2**). Out of these 12 SNPs, SNP AX-95078558 on Chr7B was associated with five traits namely, GFD, DM, TGW, PHT, and HI, whereas another SNP AX-94833876 (Chr2D) was associated with three traits, GFD, DM, and PHT. The remaining ten SNPs were associated with two traits each. AX-94779369 (Chr4D) and AX-94853198 showed association with GFD and DM; AX-94449793 and AX-95207464 were associated with GNM and PTL, and AX-95012948 (Chr1B), AX-95099434 (Chr3A), AX-94481464 (Chr2B), AX-94508364 (Chr2B), AX-94616006 (Chr7A), and AX-95104956 (Chr3B) were associated with GY and GFR (**Table 2**).

Besides multi-trait SNPs, some genomic regions contained QTLs for more than one trait. The genomic regions harboring QTLs for more than one trait included Chr1DS (27–31.69 Mb), two QTLs for GY and one QTL for HI; Chr2BS (184–205 Mb), two QTLs each for GFR and GY, and Chr2DS (30.2–32.2 Mb), one QTL each for DM, GFD, PTL and PHT. The pair-wise LD among the clustered SNPs was low, and so these were considered independent QTLs.

Favorable alleles: We have also examined promising heat tolerant lines for the presence of favorable alleles of QTLs identified in the present study. Most of the heat tolerant accessions possessed favorable alleles for the majority of QTLs associated with the analyzed traits. The accessions where favorable alleles were present for maximum number of traits were RAJ403 (GY, TGW, GFR, PTL, BM) followed by HD2888 (GY, TGW, GFR, PTL, BM), WH1142 (GY, HI, GFR, PTL, BM), and 11-F1-3 (GY, PHT, TGW, GFR, GNM). The accessions with highest number of favorable alleles were RAJ4083 (24) followed by HD2932 (22) and WH1142 (22). Moreover, for the trait PTL, 12 favorable alleles were present in HD2932 followed by RAJ403 (11), and DBW93 (10) whereas six favorable alleles were present in DBW93 and HD3118 for the GFR trait. However, a maximum of four favorable alleles was found in IC416188, IC443636, IC335732, IC536375, IC534306, IC252655, RAJ4083, HD2932, K8027, HD3118 followed by C306 for GY, and five favorable alleles were observed in HGP1-306, HD3118, WH1142 followed by J31-170 for HI and DBW107, C306 followed by RAJ4083 for TGW (**Table S5**). There were some lines that showed higher level of heat tolerance in all the three locations, however possessed only few of these favorable alleles (HD2888, IC611273, J31-170, and IC416188). These lines might have some other traits (biochemical/physiological) responsible for enhanced level of tolerance.

## Discussion

Wheat is one of the most important food and feed crops in the world, and terminal heat stress is the major factor affecting wheat productivity globally. Therefore, improving terminal heat tolerance for wheat cultivars will have a huge economic impact worldwide. Globally, there have been many efforts to understand physiological, genetic, and molecular basis of terminal heat stress tolerance in wheat (Cossani and Reynolds, 2012; Talukder et al., 2014). The present analysis was conducted to map QTLs and identify SNP markers associated with agronomical traits under very late sown wheat in order to accelerate MAS for development of heat tolerance wheat varieties.

The wheat AM panel used in this study showed large phenotypic variation for the majority of analyzed traits related to phenology and yield attributes. The variation was quite high specifically for DM, GFD, PHT, PTL, GNM, GW, TGW, and HI based on PCA. Genotypes were grouped into three distinct groups based on 2D plot of PCA. This may be attributed to diverse genetic background of genotypes included in the wheat AM panel. The AM panel included indigenous landraces and local germplasm collection (NP876, Baxi 489, Katha Gehun, *etc.*) that are well adapted to diverse climatic conditions and possess traits that might confer adoption to abiotic stresses including heat stress. Various other studies have reported large variations in agronomic traits in the diverse germplasm lines of wheat under heat stress conditions (Maulana et al., 2018; Qaseem et al., 2018; Qaseem et al., 2019). The large phenotypic variation in the AM panel showed that it was highly suitable for mapping heat tolerance associated traits. Identification of favorable alleles from the heat tolerant germplasm lines HD2888, IC611071, IC611273, IC75240, IC321906, IC416188, and J31-170 would facilitate their targeted introgression into popular wheat cultivars. The tolerant genotypes HD2888 and IC75240 both have C306 (a popular drought tolerant wheat cultivar released in 1960’s) in their parentage. J31-170 has the heat tolerant variety Raj3765 as one of the parents, whereas the IC321906 is a local line from the rainfed area of Pakur located in the Jharkhand state of India.

In our analysis, heritability values were high for many analyzed traits which indicate uniformity in the performance of genotypes across the years and locations. These were in the similar range as reported by Paliwal et al. (2012) and Qaseem et al. (2019). Correlation analysis revealed GY was positively correlated with GFR, DM, BM, GFD, TGW, and HI under late sown conditions. Similar trends have also been recorded earlier for most of these traits under heat stress condition (Paliwal et al., 2012; Bhusal et al., 2017). This suggests that GY and its positively correlated traits such as, GFR, GFD, and TGW can be selected to identify heat tolerant genotypes. Further, in this analysis, we have ranked genotypes based on GGE biplot analysis which takes into account the genotype–environment interaction. The germplasm lines IC598225, IC252897, IC445595, IC543356, and IC539565 were the highest ranked for GY under late sown conditions. These lines could be the potential sources for transferring terminal heat tolerance in popular wheat cultivars.

LD is one of the most important factors that determine the power of marker trait association analysis. We observed faster decay of LD in the subgenome A than in the other two subgenomes (B and D). Many other studies have showed rapid decay of LD in subgenome A (Chao et al., 2010; Voss-Felsa et al., 2015). In our analysis, the longest LD decay distance was observed for the D subgenome as reported in previous studies (Chao et al., 2010; Voss-Felsa et al., 2015; Maulana et al., 2018; Qaseem et al., 2019). It should be noted that the LD decay is influenced by population composition and could vary in different populations, but broadly the subgenome D has longer LD decay distance as compared to the other two subgenomes.

Although GWA analysis enables high resolution mapping of targeted traits, it might also reveal spurious associations if the confounding factors such as population structure and genetic-relatedness among individuals are not accounted for. Therefore, in our analysis, a MLM based method which accounts both these factors was used (Zhang et al., 2010). The population structure analysis using model-based approach, N-J based phylogeny, and PCA revealed three subpopulations in the AM panel. However, the observed clustering pattern was not explained on the basis of geographical distribution of the included germplasm lines. One of the possible reasons for this could be extensive sharing of germplasm within the wheat breeding program of India in the past six decades. The significantly high proportion of admixture in the three subpopulations based on STRUCTURE analysis confirms extensive intermixing of the genomes among the AM panel genotypes. An earlier study (Singh et al., 2018) has also reported high percentage of admixtures in the Indian germplasm lines. Nevertheless, we did observe that advance breeding lines of two different crosses were grouped as expected. Eleven lines from cross RAJ3765/P11632 were grouped in subpopulation I whereas fifteen lines derived from cross HD2808/HUW510 were grouped in subpopulations II. This suggests that 35K SNP markers could efficiently grouped the genotypes based on their genetic makeup.

Genome-wide association analysis revealed a total of 69 QTLs (10 significantly associated and 59 suggestive) for 10 agronomical traits including PTL, DM, BM, GFD, GNM, GY, GFR, PHT, TGW, and HI recorded under late sown conditions. Surprisingly, a large number of QTLs (17 suggestive QTLs) were associated with PTL. PTL is the count of the tillers that produces spikes and seeds and is thus considered a key determinant of wheat yield under abiotic stress conditions such as heat and drought. For this reason, genes controlling PTL are among the most important targets for yield improvement under heat stress conditions (Li et al., 2010; Hendriks et al., 2015). The 17 suggestive QTLs for PTL were distributed on seven chromosomes, Chr1A (two QTLs), Chr2D (three QTLs), Chr5B (one QTL), Chr6B (two QTLs), Ch7A (two QTLs), Ch7B (three QTLs), and Ch7D (four QTLS). Previous studies have reported QTLs for PTL on several chromosomes including Chr1B, Chr3B, Chr4B, Ch6B, Ch7A, and Ch7D (Quarrie et al., 2005; Naruoka et al., 2011; Wang et al., 2016; Liu et al., 2018; Ren et al., 2018). Naruoka et al. (2011) reported a major QTL for PTL on the long arm of Chr6B which was consistent across environments explaining 9–17% of phenotypic variation for PTL. Our analysis also revealed a QTL for PTL on the long arm of Chr6B in the same region which confirms involvement of this genomic region in determining PTL. The QTL on the short arm of Chr6B and Chr5B may represent novel QTL as there is no previous report of QTL for this trait on both these chromosomes. Further, the presence of many QTLs for PTL under late sown conditions suggests that many other QTLs in addition to those involved in tiller development under normal condition may be playing an important role.

The abiotic stress conditions particularly drought and heat stress cause reduction in PHT which in turn affect effective source for photosynthate development and thus to various agronomical and yield related traits. This study, revealed as many as 12 QTLs (two significantly associated and ten suggestive) for PHT. Two significantly associated QTLs were present on Chr2D and Chr3B. The significantly associated QTL on Chr2D is located on its short arm and in the same genomic region where the semi-dwarfing gene *Rht8* was previously reported, suggesting that some of the exotic germplasm lines included in our analysis carried this gene. Unlike the *Rht1* and *Rht2*, this semi-dwarfing gene is GA (gibberellic acid) sensitive and has shown to reduce wheat plant height in the hot and dry environment without any yield penalty (Kowalski et al., 2016). The QTL on Chr3B is located on its short arm and coincides with genomic region that contained *Rht5* gene responsible for reduced plant height (Ellis et al., 2005; Daoura et al., 2014). Association of *Rht5* and *Rht8* with PHT in our study suggests that dwarfing related genes could be a crucial role in heat tolerance as they could affect expression of various yield related traits. Further, Two QTLs for PHT were identified on Chr2A (one each on the long and short arm) and were found to be located in the same genomic regions where QTL for PHT were reported in a previously study (Qaseem et al. (2019). A QTL for PHT on Chr4D was located on the long arm, and essentially corresponds to a previously mapped locus for PHT (Zanke et al., 2014). Since it is known that reduction in PHT influences a range of other agronomic traits, such as heading date, fertile tiller number, spike length and number, it may be hypothesized that the novel PHT associated genomic regions and particularly few well-known *Rht* loci detected in this study may be playing a role in enhancing adoptions and yield in heat stress.

GY is the most desirable agronomic trait; however, its genetic regulation is very complex, controlled by a number of genes whose expressions are influenced by environmental conditions (Bennett et al., 2012; Ain et al., 2015). Our analysis revealed 14 QTLs (two significantly associated and 12 suggestive) for GY, distributed on Chr3A, Chr7A, Chr1B, Chr2B, Chr3B, Chr5B, Chr1D, and Chr3D. Out of these 14 GY associated QTLs, four QTLs, one each on Chr7A, Chr2B, Chr3B, and Chr6B were consistently found at two locations and could be tested for their utility in other wheat growing environments as well. Earlier studies have also identified QTLs for GY on Chr7A, Chr1B, Chr2B, and Chr3B under heat stress (Pinto et al., 2010; Bennett et al., 2012; Acuña-Galindo et al., 2015). Moreover, the two identified QTLs on the short arm of Chr1B and one QTL on the short arm of Chr1D were located in almost the same genomic region reported to harbor meta-QTLs (M-QTLs); M-QTL2 and M-QTL9 for GY under drought and heat stress conditions (Acuña-Galindo et al., 2015). Since these meta-QTLs were identified by collating information from a large number of studies, GY associated QTLs (Chr1B and Chr1D) identified in our analysis represent reliable QTLs. The two most significantly associated QTLs for GY, one on the long arm of Chr3A and another on the short arm of Chr7A appear to be the novel QTLs under heat stress conditions. Further, among the GY component traits, TGW is the most important for which three significant QTLs were identified, one each on Chr2BL, Chr5BL, and Chr7BL. The TGW associated QTL identified on Chr5BL falls almost in the same region where a M-QTL for TGW was reported under heat and drought conditions (Acuña-Galindo et al., 2015). Paliwal et al. (2012) have also reported QTLs for TGW on Chr2BL and Chr7BL under heat stress conditions. These TGW associated genomic regions may be targeted for yield improvement under heat stress conditions.

GFR is an important determinant of heat tolerance in wheat (Kumar et al., 2017). Under late sowing, GFD is shortened which affects grain filling, thereby causing a drastic reduction in yield. In this context, genotypes with high GFR rate can synthesize an ample quantity of photosynthates required for optimum grain development. However, despite the importance of GFR in conferring heat tolerance in wheat genotypes, its genetic basis is not well understood, and there is scant information on QTLs controlling this trait (Bhusal et al., 2017). Our analysis revealed six QTLs for GFR (five significantly associated and one suggestive) distributed on Chr3A, Chr3B, Chr7A, and Chr2B. Previously genomic regions for GFR were identified on Chr1A, Chr2A, Chr6A, Chr6B, and Chr7D (Bhusal et al., 2017). Therefore, all the GFR associated QTLs identified in the present study represent novel genomic regions for this very important trait under heat stress conditions.

HI is an important yield associated trait which determines the performance of wheat genotypes under abiotic stress conditions such as heat and drought. Previous studies have identified QTLs for HI on Chr1A, Chr2B, Chr5B, and Chr6B (Assanga et al., 2017; El-Feki et al., 2018). In our analysis, three significantly associated QTLs for HI were identified, one QTL on Chr1DS and other two QTLs on 7BL.The significant HI associated QTLs identified in this study are novel and need further validation for use in MAS.

GNM is another component of yield, which is severely affected due to accelerated physiological maturity under late sown conditions. Though a good number of QTLs have been identified for grain number per spike under heat stress, there exists limited information on genomic regions/genes associated with GNM. Our study revealed three novel genomic regions for GNM on Chr5B, Chr1D, and Chr7B. A QTL for grain number on Chr5B but on its long arm was identified by Pinto et al. (2010).

All genomic regions identified in this study should be validated in an independent biparental mapping population between parents with opposite alleles and also be tested for stability under many environmental conditions before they could be exploited in wheat breeding programs through MAS.

Validation is of most interest for the total of twelve SNPs/genomic regions that were associated with two or more agronomical traits, suggesting that genomic regions controlling two or more desirable agronomical traits might have been selected during wheat domestication. Interestingly, one of these was associated with five traits GFD, DM, TGW, PHT, and HI. Co-localization of QTLs for agronomical and grain related traits has been demonstrated in earlier studies as well (Bhusal et al., 2017). This is considered very useful from the utilization perspective, as the markers can be used to select multiple yield component traits (QTL) in breeding programs.

Two wheat germplasm lines (IC75240 and IC321906) that had few favorable alleles for the QTLs identified in this study but showed a high level of heat tolerance might be because of the presence of other favorable alleles/genes which could not be identified in the present study. Hence, some other traits related to heat tolerance may also be explored in addition to the ten traits explored in the present investigation. Our study has provided novel genomic regions for various heat tolerant associated traits These must be validated through other approaches such as biparental mapping and functional genomics to define their role, as it has been previously reported in various studies that the GWAS approach may result in false positive associations which require further validation (Tam et al., 2019) prior to the use for marker assisted breeding

In conclusion, our study provided an insight into the genetic architecture of 10 agronomic traits in wheat under late sown conditions. The wheat association panel used in this analysis revealed wide variability for most of the agronomic traits under late sown conditions. Some stable genotypes for late sown conditions were also identified. Genotyping using a high density 35 K array, revealed a large number of SNPs that could be used for the GWAS analysis. In total, 69 QTLs were identified for the ten agronomic traits, which included some novel genomic regions. The potential loci/QTLs and genotypes identified in this study may be further validated to determine their use in wheat breeding programs for development of heat tolerant wheat genotypes.

## Data Availability Statement

The original contributions presented in the study are included in the article/**Supplementary Files**; further inquiries can be directed to the corresponding authors.

## Author Contributions

SundK: Conceptualization, data curation, formal analysis, investigation, drafted the manuscript. JK: Investigation, data curation, data analysis. NBh: Investigation, data recording. AP: Performed genomic predictions. NBu: Analyzed the data, manuscript writing. DM: Analyzed the data, manuscript writing. DC: Analyzed the data. SuneK: Formal analysis, revised and edited the manuscript. AS: Formal analysis, scientific inputs for manuscript preparation, revised and edited the manuscript. MR: Conceptualization, data curation, formal analysis, investigation. GS: Writing—review and editing. KS: Writing—review and editing. SS: Conceptualization, data curation, formal analysis, investigation, revised the manuscript.

## Funding

Financial assistance from USAID through CIMMYT, Mexico under ARCADIA—CIMMYT—USAID project Development of heat tolerant wheat for South Asia (Grant No. OAAA-13-00001).

## Conflict of Interest

The authors declare that the research was conducted in the absence of any commercial or financial relationships that could be construed as a potential conflict of interest.
