# Supplementary material for: Genome-Wide Association Study Reveals Genomic Regions Associated With Ten Agronomical Traits in Wheat Under Late-Sown Conditions
Source: Front Plant Sci. 2020 Sep 17;11:549743. doi: 10.3389/fpls.2020.549743 (PMC7527491; doi:10.3389/fpls.2020.549743)
Supplement: Supplementary file 1 [file DataSheet_1.docx]

**Supplementary information**

Table S1: Details of the genotypes included in the present study

Table S2: Descriptive statistics and heritability of 16 evaluated traits.

TableS3: Chromosome wise**-**distribution pattern of 11,911 SNPs and the intra-chromosomal estimated LD among 205 wheat genotypes.

Table S4: Details of QTLs identified for 10 different traits at Delhi, Hisar and Karnal

Table S5: Favorable alleles of identified QTLs in some of the promising heat tolerant accessions.

Fig. S1: Frequency distribution of 16 morphological yield and quality related traits using BLUP values, (a) DH,(b) DA,(c) DM,(d) GFD,(e) PHT,(f) PTL,(g) GN,(h) GNM,(i) GW,(j) TGW,(k) BM,(l) GY, (m) HI,(n) CFL,(o) CMS,(p) GFR used for association mapping in current study.

Fig. S2: Principal component analysis (PCA) of 205 genotypes evaluated under extremely heat stress (a). 2D Plot based on 16 studied traits clustering genotypes into three groups, (b). Plotting of genotypes based on 1000 grain weight variation, (c). Plotting of genotypes based on grain filling duration.

Fig. S3: Which-won-where’, ‘mean vs. stability’ and ‘raking genotypes’ view of the GGE biplot of (a) PTL (b) GFD and (c) GN of 205 diverse wheat genotypes evaluated in three environments during season 2015-2016-2017.

Fig. S4: Which-won-where’, ‘mean vs. stability’ and ‘raking genotypes’ view of the GGE biplot of (a) GY(b) TGW and (c) HI of 205 diverse wheat genotypes evaluated in three environments during season 2015-2016-2017.

Fig. S5: LD decay across the (a) A, (b) B, (c) D sub-genomes and (d) whole genome of wheat. The values on the Y-axis represent the squared correlation coefficient (r 2) and the values at X-axis represent physical distance (Mb).

Fig.S6: Manhattan plots for five different traits (a) GFD, (b) BM, (c) TGW, (d) PTL and (e) GNM. X-axis shows position of SNP markers on 21 chromosomes (1A to 7D) and Y-axis shows significance of the association tests on a −log scale. Horizontal line represents the threshold (P <0.0001) above which markers were considered significantly associated. Manhattan plots were generated in the qqman R package, v0.1.4 (<https://cran.r-project.org/web/packages/qqman/index.html>)

Fig. S7: 10 traits Q-Q plot (a) GFD, (b) DM, (c) BM, (d) TGW, (e) PTL, (f) PHT, (g) HI, (h) GY, (i) GNM, (j) GFR.

| **Table S1:** | | | | |
| --- | --- | --- | --- | --- |
| SL NO | Entry | Alternate ID/Pedigree | Source | sub-population assigned based on STRUCTURE program |
| 1 | IC82460 | S-227 | NBPGR, New Delhi | 3 |
| 2 | EC190943 | W-223 | CIMMYT, MEXICO | 2 |
| 3 | IC531970 | HW-3083 | IARI, RS, Wellington | 2 |
| 4 | IC111840 | HP-1286 | IARI, RS, Pusa, Bihar | 1 |
| 5 | IC335685 | EGPSN(3)-73 | NBPGR, New Delhi | 2 |
| 6 | HTW 29 | HD2808/HUW510-180 | IIWBR, Karnal, Haryana | 2 |
| 7 | IC445498 | ET-95392 | IIWBR, Karnal, Haryana | 2 |
| 8 | IC290188 | Local germplasm | NBPGR, New Delhi | 2 |
| 9 | IC138428 | KA-9022 | Kanpur, Uttar Pradesh | 3 |
| 10 | IC416073 | PAU - 493/ W 8061 | Ludhiana, Punjab | 3 |
| 11 | PI-324475 | NIPHAD-4 | USDA, Maryland, USA | 1 |
| 12 | IC252869 | NW-1043 | NDUAT, Faizabad | 1 |
| 13 | IC252871 | P-10950 | NBPGR, New Delhi | 1 |
| 14 | IC290066 | Local germplasm | NBPGR, New Delhi | 2 |
| 15 | IC547662 | 9th-EGPSN-129 | NBPGR, New Delhi | 1 |
| 16 | IC335711 | EGPYT (IST)-18 | IIWBR, Karnal, Haryana | 3 |
| 17 | IC543417 | 3RD IAT-43 (ET-8548) | NBPGR, New Delhi | 2 |
| 18 | IC445595 | UP-2338 (UP 368/VL 421/UP 262) | GBPUA&T, Pantnagar | 3 |
| 19 | IC290192 | Local germplasm | NBPGR, New Delhi | 1 |
| 20 | IC335706 | EGPYT(IST)-13 | IIWBR, Karnal, Haryana | 3 |
| 21 | IC543356 | 6TH EGPSN-4 (ET-906) | NBPGR, New Delhi | 3 |
| 22 | IC328433 | SAW/GML-2 | Kathua, Jammu and Kashmir | 3 |
| 23 | IC252897 | PBW-450 | Ludhiana, Punjab | 2 |
| 24 | IC279537 | KHH-336 | TehriGarwal, Uttarakhand | 3 |
| 25 | IC535176 | WON-D-14 | NBPGR, New Delhi | 2 |
| 26 | IC335534 | HW 2045 | IARI-RS, Wellington | 3 |
| 27 | IC539565 | GW-2001-52 | IIWBR, Karnal, Haryana | 2 |
| 28 | IC539276 | HS-445 | IARI RS, Shimla | 2 |
| 29 | IC335523 | GW 322 (PBW 173/GW 196) | GAU, Vijapur, Gujarat | 3 |
| 30 | EC498424 | SERI.1B/KAUZ/HEVO/3/AMAD | CIMMYT, Mexico | 2 |
| 31 | IC252727 | HUW 478 | BHU, Varanasi | 2 |
| 32 | IC402054 | K-20008 | Haryana | 2 |
| 33 | IC335742 | SAWSN(17)-14 | IIWBR, Karnal, Haryana | 2 |
| 34 | EC425303 | BL 2066 | CIMMYT, Nepal | 3 |
| 35 | IC393882 | WH533/VEE# #5 | CCSHAU, Hissar | 3 |
| 36 | IC128684 | RAJ3476 | Jaipur, Rajasthan | 3 |
| 37 | IC535188 | WYRGP-3 | NBPGR, New Delhi | 1 |
| 38 | IC104559 | CH-26-1 | Chittorgarh,Rajasthan | 3 |
| 39 | IC536060 | WL 2206 | Others | 3 |
| 40 | IC335524 | GW-326 | GAU, Vijapur, Gujarat | 2 |
| 41 | IC252642 | HDR-92 | Unknown | 2 |
| 42 | IC333095 | NKD/YSR-2910; DL - 803 | Barwani,Madhya Pradesh | 3 |
| 43 | IC531833 | DL-96-8 | IIWBR, Karnal, Haryana | 3 |
| 44 | IC539156 | EIGN-I-(04-05)/63 | Karnal, Haryana | 3 |
| 45 | IC539600 | CLN-4 | IIWBR, Karnal, Haryana | 3 |
| 46 | IC415978 | PAU - 398; W 4900 | Ludhiana, Punjab | 2 |
| 47 | IC290156 | Local germplasm | NBPGR, New Delhi | 2 |
| 48 | IC252389 | BL-1970 | Uttar Pradesh | 3 |
| 49 | IC536168 | WL 5262 | PAU, Ludhiana | 2 |
| 50 | IC290068 | Local germplasm | NBPGR, New Delhi | 2 |
| 51 | IC543388 | WON-D-03-4 (ET-9) | NBPGR, New Delhi | 2 |
| 52 | IC279335 | HS-104 | Shimla, Himachal Pradesh | 3 |
| 53 | PI-430083 | W-212 | USDA, Maryland, USA | 3 |
| 54 | IC398010 | YS/RC-199; Kanak | Bilaspur /Himachal Pradesh | 3 |
| 55 | EC574390 | 8420 | NBPGR, New Delhi | 3 |
| 56 | EC190950 | Cultivar NO.-14 | CIMMYT, MEXICO | 3 |
| 57 | IC549449 | RAJ-4027 | Rajasthan | 3 |
| 58 | IC547561 | GW366 | Gujarat | 3 |
| 59 | IC524299 | HW 2012 | Wellington/Tamil Nadu | 1 |
| 60 | IC290196 | HW-2045; Kaushambi | IIWBR, Karnal, Haryana | 3 |
| 61 | IC598226 | DBW 93 ((Wheat x Tukuru) x Wheat) | IIWBR, Karnal, Haryana | 2 |
| 62 | HD 2932 | HD-2932 | IARI, New Delhi | 3 |
| 63 | IC547558 | RAJ-4083 | IIWBR, Karnal, Haryana | 3 |
| 64 | HD 2888 | HD-2888; Pusa Wheat -107 | IARI, New Delhi | 1 |
| 65 | K 8027 | K-8027; MAGHAR | Kanpur, Uttar Pradesh | 3 |
| 66 | IC145428 | HUW-319 | BHU, Varanasi | 3 |
| 67 | IC611071 | HD 3118/Pusa (ATTILA*2/PBW65//WBLL1*2/TUKURU) | IARI, New Delhi | 2 |
| 68 | IC611273 | DBW 107 (TUKURU x INQLAAB 91) | IIWBR, Karnal, Haryana | 2 |
| 69 | IC554661 | WH 1021 | Hisar, Haryana | 2 |
| 70 | IC589772 | PBW 644 (PBW 175/HD 2643) | PAU, Ludhiana | 2 |
| 71 | IC598225 | DBW 90 (HUW 468 x WH 730) | IIWBR, Karnal, Haryana | 2 |
| 72 | IC586642 | HI 1563 (Pusa Prachi) | Indore, Madhya Pradesh | 2 |
| 73 | IC335712 | EGPYT(IST)-20 | IIWBR, Karnal, Haryana | 2 |
| 74 | IC611479 | WH 1142 [(Chen x Aegilopssquarrosa) x (FCT x Weaver)] | Hisar, Haryana | 2 |
| 75 | IC75240 | C306/(Regent 1974` x Ch 23`) or RGN/CSK3//2*C591/3/C217/N14//C281 | Uttar Pradesh | 2 |
| 76 | EC574442 | 8483 | NBPGR, New Delhi | 2 |
| 77 | IC532037 | MP-4010 | JNKVV, Jabalpur | 3 |
| 78 | IC534596 | PI-266874 | NBPGR, New Delhi | 1 |
| 79 | IC28658 | 300; collection | Banaskantha, Gujarat | 1 |
| 80 | IC28665 | 358; collection | Kutch, Gujarat | 1 |
| 81 | IC78856 | K-3292 | Kanpur, Uttar Pradesh | 1 |
| 82 | IC128335 | LGM-227 | Jaipur, Rajasthan | 1 |
| 83 | IC416188 | PAU-608 | PAU, Ludhiana, Punjab | 2 |
| 84 | IC443636 | HW-2017 | IIWBR, Karnal, Haryana | 3 |
| 85 | IC128573 | KA9079 | Kanpur, Uttar Pradesh | 3 |
| 86 | IC445365 | 7th EGPSN-78 | IIWBR, Karnal, Haryana | 3 |
| 87 | IC335732 | HRWSN(10)-171 | IIWBR, Karnal, Haryana | 2 |
| 88 | IC128218 | KRLI-4 | Kanpur, Uttar Pradesh | 3 |
| 89 | IC539602 | CLN-6 | IIWBR, Karnal, Haryana | 2 |
| 90 | IC536375 | RAJ-3268 | Jaipur, Rajasthan | 3 |
| 91 | IC535706 | PBW126 | Ludhiana, Punjab | 3 |
| 92 | IC36761 | Collection | Jagheri, Shimla, Himachal Pradesh | 1 |
| 93 | IC290155 | Local germplasm | NBPGR, New Delhi | 3 |
| 94 | IC321906 | C-306 variant | Pakur, Jharkhand | 2 |
| 95 | IC296681 | Hindi 62; Local germplasm | IIWBR, Karnal, Haryana | 1 |
| 96 | 11-F1-16 | RAJ4014/HUW510 | IIWBR, Karnal, Haryana | 3 |
| 97 | 11-F1-3 | K7903/P11632 | IIWBR, Karnal, Haryana | 3 |
| 98 | 11-F1-8 | HD2808/RAJ4014 | IIWBR, Karnal, Haryana | 3 |
| 99 | 11-F1-2 | K7903/RAJ4014 | IIWBR, Karnal, Haryana | 1 |
| 100 | J31-73 | RAJ3765/P11632-73 | IIWBR, Karnal, Haryana | 3 |
| 101 | J31-145 | RAJ3765/P11632-145 | IIWBR, Karnal, Haryana | 3 |
| 102 | J31-33 | RAJ3765/P11632-33 | IIWBR, Karnal, Haryana | 3 |
| 103 | IC539599 | CLN-3 | IIWBR, Karnal, Haryana | 3 |
| 104 | J31-23 | RAJ3765/P11632-23 | IIWBR, Karnal, Haryana | 3 |
| 105 | J31-83 | RAJ3765/P11632-83 | IIWBR, Karnal, Haryana | 3 |
| 106 | J31-170 | RAJ3765/P11632-170 | IIWBR, Karnal, Haryana | 3 |
| 107 | F1-5 | HD2808/RAJ4014 | IIWBR, Karnal, Haryana | 3 |
| 108 | J31-80 | RAJ3765/P11632-80 | IIWBR, Karnal, Haryana | 3 |
| 109 | J31-102 | RAJ3765/P11632-102 | IIWBR, Karnal, Haryana | 3 |
| 110 | J31-2 | RAJ3765/P11632-2 | IIWBR, Karnal, Haryana | 3 |
| 111 | J31-101 | RAJ3765/P11632-101 | IIWBR, Karnal, Haryana | 3 |
| 112 | J31-165 | RAJ3765/P11632-165 | IIWBR, Karnal, Haryana | 3 |
| 113 | HGP1-318 | HD2808/HUW510-318 | IIWBR, Karnal, Haryana | 3 |
| 114 | IC112243 | Raj-3550 | Jaipur, Rajasthan | 3 |
| 115 | HGP1-306 | HD2808/HUW510-306 | IIWBR, Karnal, Haryana | 2 |
| 116 | HGP1-460 | HD2808/HUW510-460 | IIWBR, Karnal, Haryana | 2 |
| 117 | HGP1-403 | HD2808/HUW510-403 | IIWBR, Karnal, Haryana | 2 |
| 118 | HGP1-32 | HD2808/HUW510-32 | IIWBR, Karnal, Haryana | 2 |
| 119 | HGP1-468 | HD2808/HUW510-468 | IIWBR, Karnal, Haryana | 2 |
| 120 | HGP1-470 | HD2808/HUW510-470 | IIWBR, Karnal, Haryana | 2 |
| 121 | HGP1-435 | HD2808/HUW510-435 | IIWBR, Karnal, Haryana | 3 |
| 122 | HGP1-180 | HD2808/HUW510-180 | IIWBR, Karnal, Haryana | 3 |
| 123 | IC290292 | Local germplasm | NBPGR, New Delhi | 3 |
| 124 | HGP1-359 | HD2808/HUW510-359 | IIWBR, Karnal, Haryana | 3 |
| 125 | IC 138852 | NKG/92 (NIC-8835) | Kinnaur, Himachal Pradesh | 2 |
| 126 | HGP1-107 | HD2808/HUW510-107 | IIWBR, Karnal, Haryana | 2 |
| 127 | HGP1-315 | HD2808/HUW510-315 | IIWBR, Karnal, Haryana | 2 |
| 128 | HGP1-448 | HD2808/HUW510-448 | IIWBR, Karnal, Haryana | 2 |
| 129 | HGP1-208 | HD2808/HUW510-208 | IIWBR, Karnal, Haryana | 2 |
| 130 | HGP1-305 | HD2808/HUW510-305 | IIWBR, Karnal, Haryana | 2 |
| 131 | IC533903 | CITR-7304 | NBPGR, New Delhi | 1 |
| 132 | IC75362 | NI-8841 | MPKV RS, Piphad | 3 |
| 133 | EC190984 | Cultivar NO.-59 | CIMMYT, MEXICO | 3 |
| 134 | IC290246 | Local germplasm | NBPGR, New Delhi | 2 |
| 135 | IC416141 | PAU - 561 (W 9301) | PAU, Ludhiana | 2 |
| 136 | PI-430119 | W-238 | USDA, Maryland, USA | 1 |
| 137 | EC11129 | CI 12100 / PI139599 | USDA, USA | 1 |
| 138 | IC60221 | Collection | NBPGR, New Delhi | 1 |
| 139 | IC290143 | Local germplasm | NBPGR, New Delhi | 2 |
| 140 | IC252517 | DL-975-1 | West Bengal | 3 |
| 141 | IC112049 | AO-90; collection | NBPGR, New Delhi | 1 |
| 142 | IC416089 | PAU - 509 (W 8171) | Ludhiana, Punjab | 2 |
| 143 | IC533748 | B-3-C-1; collection | NBPGR, New Delhi | 3 |
| 144 | IC574474 | KRL 210 | Karnal, Haryana | 2 |
| 145 | EC575006 | 9197 | NBPGR, New Delhi | 2 |
| 146 | IC396586 | DPY-503 (Gehun Katha) | Pali, Rajasthan | 3 |
| 147 | IC401996 | RAJ-4012 | Jaipur, Rajasthan | 3 |
| 148 | IC536468 | K-8415 | CSA, Kanpur, Uttar Pradesh | 2 |
| 149 | IC533717 | 4-EGPSN-132 | NBPGR, New Delhi | 3 |
| 150 | IC335701 | EGPYT(IST)-6 | IIWBR, Karnal, Haryana | 2 |
| 151 | IC138617 | NP-876 | Hoshangabad, Madhya Pradesh | 1 |
| 152 | EC425336 | BL 2031 | CIMMYT, NEPAL | 2 |
| 153 | IC290076 | Collection | NBPGR, New Delhi | 2 |
| 154 | IC335761 | SAWSN(17)-61 | IIWBR, Karnal, Haryana | 2 |
| 155 | IC111905 | A-557; collection | NBPGR, New Delhi | 3 |
| 156 | IC335669 | EGPSN(3)-18 | IIWBR, Karnal, Haryana | 2 |
| 157 | IC531121 | WL 7194 | PAU, Ludhiana | 3 |
| 158 | IC252813 | K-9644 (HD 2402/K 8305) | Kanpur, UP | 3 |
| 159 | HTW26 | - | Karnal, Haryana | 2 |
| 160 | IC543419 | 10TH HTWYT-4 (ET-8) | NBPGR, New Delhi | 2 |
| 161 | IC335690 | EGPSN(3)-117 | IIWBR, Karnal , Haryana | 2 |
| 162 | IC539292 | PSR-11380(AMB) | Karnal, Haryana | 1 |
| 163 | IC145243 | BAXI-489 | Unknown | 2 |
| 164 | IC335677 | EGPSN(3)-55 | IIWBR, Karnal, Haryana | 2 |
| 165 | IC252816 | KRL-13 | Karnal, Hryana | 1 |
| 166 | IC303071 | RAJ-3777(Raj 3160/HD 2449) | Jaipur, Rajstahn | 3 |
| 167 | IC290323 | Collection | NBPGR, New Delhi | 2 |
| 168 | IC252415 | BW-267 | West Bengal | 2 |
| 169 | IC128184 | HDR-77 (PTZ/2*HD2204) | IIWBR, Karnal, Haryana | 3 |
| 170 | IC252960 | VL-688 | VPKAS, Almora | 3 |
| 171 | IC75242 | HD-2329 (HD-1962/E 4870/3/ K 65/5/SKA/6/UP262) | IARI, New Delhi | 3 |
| 172 | IC290225 | K-9241 | Shimla/Himachal Pradesh | 3 |
| 173 | IC542901 | PAU- 491 | PAU, Ludhiana | 1 |
| 174 | IC128523 | KA9008 | CSA, Kanpur, Uttar Pradesh | 2 |
| 175 | IC104562 | CH-27-1 | Chittorgarh, Rajasthan | 2 |
| 176 | EC190899 | Cultivar NO.-37 | CIMMYT, MEXICO | 2 |
| 177 | IC290080 | Local germplasm | NBPGR, New Delhi | 2 |
| 178 | IC534306 | Local germplasm | NBPGR, New Delhi | 1 |
| 179 | IC336741 | VR-13 | Dhar,Madhya Pradesh | 3 |
| 180 | IC128641 | KA90171 | Kanpur,Uttar Pradesh | 3 |
| 181 | IC539137 | EIGN-I-(04-05)/33 | Karnal, Haryana | 2 |
| 182 | IC543293 | PAU-1218 | PAU, Ludhiana | 2 |
| 183 | IC111888 | Local germplasm | NBPGR, New Delhi | 1 |
| 184 | IC252690 | HS-295 | Shimla, Himachal Pradesh | 2 |
| 185 | EC463382 | KRL 1-4 | Karnal, Haryana | 2 |
| 186 | IC335792 | KAUZ+1B.1R | CIMMYT, MEXICO | 2 |
| 187 | IC443704 | GW-349 | Karnal, Haryana | 3 |
| 188 | EC190878 | Cultivar NO.-8 | CIMMYT, MEXICO | 3 |
| 189 | IC416075 | PAU - 495 (W 8067) | Ludhiana, Punjab | 2 |
| 190 | IC252472 | CDWR-9513 | West Bengal | 2 |
| 191 | IC539155 | EIGN-I-(04-05)/62 | Karnal, Haryana | 2 |
| 192 | IC252655 | HI-1456 | IARI, RS, Indore | 3 |
| 193 | IC290173 | Local germplasm | NBPGR, New Delhi | 3 |
| 194 | EC576555 | E-3129 | Unknown | 2 |
| 195 | IC252650 | HI 1384 | IARI RS, Indore | 2 |
| 196 | IC252509 | DL-230-5 | West Bengal | 3 |
| 197 | IC566223 | HPW42/CAPN2032/UNATH K.S. | Tutikandi, Shimla | 3 |
| 198 | IC534324 | PI-176242/SIRKA/K-52915 | Kumaon, Uttarakhand | 2 |
| 199 | IC533761 | B-28-C-1; collection | NBPGR, New Delhi | 3 |
| 200 | IC443694 | PBW-527 | IIWBR, Karnal, Haryana | 3 |
| 201 | IC416146 | PAU - 566 (W 9390) | Ludhiana, Punjab | 2 |
| 202 | EC574731 | 8832 | NBPGR, New Delhi | 2 |
| 203 | IC111845 | HD-2236 | IARI, New Delhi | 3 |
| 204 | EC609554 | 03ABIO-05-D02-H037 | South Perth, Australia | 2 |
| 205 | IC252414 | BW-1050 | West Bengal | 2 |

| **Table S2:** | | | | |  |  |  |
| --- | --- | --- | --- | --- | --- | --- | --- |
|  | DELHI | | HISAR | |  | KARNAL |  |
| TRAIT | RANGE | MEAN+SE | RANGE | MEAN+SE | RANGE | MEAN+SE | HERITABILITY |
| DH | 48-77 | 65.40 + 0.35 | 57-82 | 69.75+0.34 | 60-82 | 69.39+ 0.36 | 0.91 |
| DA | 53-82 | 69.83+0.35 | 65-87 | 74.45+0.32 | 64-88.75 | 73.74+0.40 | 0.90 |
| DM | 77.5-114 | 100.00+0.33 | 96-122 | 104.64+0.41 | 90-120 | 99.91+0.58 | 0.78 |
| GFD | 16.25-45.25 | 30.19+0.38 | 16-52 | 30.62+0.55 | 20-39 | 26.68+0.33 | 0.89 |
| PHT | 60-115 | 81.98+0.77 | 34-131.5 | 70.72+1.28 | 42-113.67 | 85.55+0.84 | 0.81 |
| PTL | 28-107 | 67.44+1.07 | 6-88 | 45.96+1.44 | 39-123 | 69.06+1.04 | 0.67 |
| GN | 19.6-60.6 | 41.05+0.60 | 22-80 | 48.40+0.82 | 20.2-64.8 | 40.75+0.60 | 0.40 |
| GNM | 1557.6-23604 | 10999.75+264.78 | 0-20826 | 8347.10+295.87 | 1423.75-22230 | 10931.40+240.40 | 0.46 |
| GW | 0.34-2.3 | 1.13+0.03 | 0.42-5.2 | 1.89+0.06 | 0.54-2.40 | 1.36+0.03 | 0.53 |
| TGW | 10.06-48.98 | 29.63+0.57 | 10.4-51.76 | 31.49+0.55 | 19.11-61.74 | 33.29+0.49 | 0.93 |
| BM | 380.03-3750 | 1504.61+51.19 | 394-2750 | 1424.24+30.09 | 872.50-4000 | 1843.83+32.24 | 0.81 |
| GY | 73.2-1300 | 346.34+15.99 | 112-985 | 374.10+11.12 | 205.25-1529 | 538.34+13.40 | 0.94 |
| HI | 10.17-80.03 | 24.30+0.77 | 10.36-52.18 | 26.95+0.63 | 14.39-48.83 | 29.19+0.40 | 0.81 |
| CFL | 0.45-0.78 | 0.64+0.004 | 0.55-0.79 | 0.69+0.003 | 0.49-0.84 | 0.77+0.003 | 0.33 |
| CMS | 23.08-95.58 | 60.54+1.02 | 12.56-76 | 46.14+1.07 | 12.25-94.59 | 51.54+1.20 | 0.86 |
| GFR | 3.14-42.43 | 11.26+0.52 | 3.64-32.83 | 12.64+0.39 | 5.74-46.33 | 20.79+0.51 | 0.87 |

| Table S3: | | | | | | |
| --- | --- | --- | --- | --- | --- | --- |
| Chr | Estimated Size (~Mb) | No. of SNP | Average number  of SNPs per MB | Chr. LD (r^2^) | D prime | No of marker pairs in perfect LD |
| 1A | 594. 1 | 708 | 1. 19 | 0. 219 | 0. 575 | 1362 |
| 1B | 689. 85 | 842 | 1. 22 | 0. 349 | 0. 704 | 3929 |
| 1D | 495. 45 | 673 | 1. 36 | 0. 341 | 0. 623 | 3435 |
| 2A | 780. 8 | 729 | 0. 93 | 0. 200 | 0. 559 | 876 |
| 2B | 801. 26 | 883 | 1. 10 | 0. 271 | 0. 676 | 947 |
| 2D | 651. 85 | 712 | 1. 09 | 0. 254 | 0. 596 | 1889 |
| 3A | 750. 84 | 488 | 0. 65 | 0. 127 | 0. 523 | 186 |
| 3B | 830. 83 | 717 | 0. 86 | 0. 184 | 0. 605 | 271 |
| 3D | 615. 55 | 406 | 0. 66 | 0. 107 | 0. 463 | 107 |
| 4A | 744. 59 | 430 | 0. 58 | 0. 143 | 0. 527 | 235 |
| 4B | 673. 62 | 320 | 0. 48 | 0. 115 | 0. 486 | 128 |
| 4D | 509. 86 | 210 | 0. 41 | 0. 078 | 0. 413 | 35 |
| 5A | 709. 77 | 545 | 0. 77 | 0. 120 | 0. 485 | 379 |
| 5B | 713. 15 | 807 | 1. 13 | 0. 177 | 0. 555 | 1134 |
| 5D | 566. 08 | 524 | 0. 93 | 0. 133 | 0. 471 | 481 |
| 6A | 618. 08 | 211 | 0. 34 | 0. 112 | 0. 446 | 133 |
| 6B | 720. 99 | 638 | 0. 88 | 0. 177 | 0. 597 | 373 |
| 6D | 473. 59 | 410 | 0. 87 | 0. 118 | 0. 473 | 169 |
| 7A | 736. 71 | 618 | 0. 84 | 0. 129 | 0. 529 | 309 |
| 7B | 750. 62 | 553 | 0. 74 | 0. 134 | 0. 521 | 189 |
| 7D | 638. 69 | 487 | 0. 76 | 0. 084 | 0. 431 | 75 |

| Table S4: | | | | | | | |
| --- | --- | --- | --- | --- | --- | --- | --- |
| Traits | Markers | Chromosome | Position | p-value | R2 (%) | Env. |  |
| BM | AX-94581232 | 2D | 65563323 | 0.000193 | 8.80 | Delhi, Karnal |  |
| BM | AX-94792644 | 7D | 285237883 | 0.000472 | 8.46 | Hisar, Karnal |  |
| DM | AX-94853198 | 5D | 379790266 | 0.000748 | 7.35 | Hisar, Karnal |  |
| GFD | AX-94384220 | 7D | 188318675 | 1.4E-05 | 10.10 | Hisar, Karnal |  |
| GFD | AX-94409188 | 2D | 488234755 | 0.000734 | 7.38 | Hisar, Karnal |  |
| GFD | AX-94430026 | 1B | 640157785 | 0.000507 | 7.88 | Delhi, Karnal |  |
| GFD | AX-94497710 | 4D | 324221156 | 0.000565 | 8.34 | Hisar, Karnal |  |
| GFD | AX-94507963 | 1B | 553651865 | 0.000931 | 7.27 | Delhi, Hisar |  |
| GFD | AX-94538863 | 6A | 6735052 | 2.29E-05 | 11.11 | Delhi, Karnal |  |
| GFD | AX-94648162 | 2B | 207058354 | 0.000115 | 11.52 | Delhi, Karnal |  |
| GFD | AX-94649755 | 5D | 546690003 | 7.05E-05 | 10.15 | Delhi, Karnal |  |
| GFD | AX-94684351 | 5B | 440237550 | 7.79E-07 | 13.70 | Delhi, Karnal |  |
| GFD | AX-94810466 | 1A | 556793974 | 0.000966 | 7.19 | Delhi, Karnal |  |
| GFD | AX-94889110 | 3B | 107666333 | 5.16E-05 | 11.94 | Delhi, Karnal |  |
| GFD | AX-94918415 | 2B | 623056105 | 7.45E-05 | 10.56 | Delhi,Hisar, Karnal |  |
| GFD | AX-94930932 | 5A | 563456261 | 3.32E-05 | 11.77 | Delhi, Karnal |  |
| GFD | AX-94955512 | 5A | 564079678 | 2.01E-05 | 12.34 | Hisar, Karnal |  |
| GFD | AX-95078558 | 7B | 652933607 | 1.76E-05 | 12.92 | Hisar, Karnal |  |
| GFD | AX-95222660 | 5B | 462127466 | 0.000149 | 9.71 | Hisar, Karnal |  |
| GFR | AX-94481464 | 2B | 205477435 | 2.77E-05 | 11.44 | Delhi, Karnal |  |
| GFR | AX-94508364 | 2B | 184800377 | 4.42E-06 | 13.24 | Delhi, Karnal |  |
| GFR | AX-94616006 | 7A | 205485005 | 1.11E-05 | 13.06 | Delhi, Karnal |  |
| GY | AX-94422821 | 1B | 98920762 | 0.000736 | 7.37 | Delhi, Karnal |  |
| GY | AX-94424335 | 7B | 561748138 | 9.83E-05 | 9.58 | Delhi, Karnal |  |
| GY | AX-94433426 | 1D | 162061526 | 0.000519 | 7.74 | Delhi, Karnal |  |
| GY | AX-94437800 | 6B | 116113554 | 0.000397 | 8.02 | Delhi, Karnal |  |
| GY | AX-94453771 | 1B | 156811480 | 0.000788 | 7.31 | Delhi, Karnal |  |
| GY | AX-94481464 | 2B | 205477435 | 1.7E-06 | 14.01 | Delhi, Karnal |  |
| GY | AX-94508364 | 2B | 184800377 | 4.64E-06 | 13.06 | Delhi, Karnal |  |
| GY | AX-94544437 | 1D | 27556030 | 0.000311 | 8.44 | Delhi, Karnal |  |
| GY | AX-94616006 | 7A | 205485005 | 5.99E-07 | 15.83 | Delhi, Karnal |  |
| GY | AX-94665409 | 1A | 313739062 | 3.59E-05 | 10.61 | Delhi, Karnal |  |
| GY | AX-94723838 | 1B | 66938107 | 3.34E-05 | 10.76 | Delhi, Karnal |  |
| GY | AX-94767447 | 3A | 150081441 | 1.13E-05 | 11.98 | Delhi, Karnal |  |
| GY | AX-94870559 | 1B | 100648488 | 0.000739 | 7.37 | Delhi, Karnal |  |
| GY | AX-94890395 | 1D | 248200443 | 3.02E-05 | 10.82 | Delhi, Karnal |  |
| GY | AX-94919771 | 1B | 150539477 | 0.000745 | 7.37 | Delhi, Karnal |  |
| GY | AX-94946002 | 2D | 17605241 | 0.000695 | 7.57 | Hisar, Karnal |  |
| GY | AX-94981856 | 1D | 464319073 | 2.1E-05 | 11.45 | Delhi, Karnal |  |
| GY | AX-94991116 | 1B | 91557695 | 1.03E-05 | 12.21 | Delhi, Karnal |  |
| GY | AX-95010687 | 2B | 74001933 | 0.000787 | 7.48 | Delhi, Karnal |  |
| GY | AX-95099434 | 3A | 434614902 | 0.000307 | 8.54 | Delhi, Karnal |  |
| GY | AX-95104956 | 3B | 684617559 | 3.99E-06 | 13.61 | Delhi, Karnal |  |
| GY | AX-95105278 | 2B | 104832853 | 8.87E-05 | 9.75 | Delhi, Karnal |  |
| GY | AX-95135390 | 1B | 99713230 | 0.000739 | 7.37 | Delhi, Karnal |  |
| GY | AX-95164232 | 5D | 389698142 | 8.1E-05 | 9.73 | Delhi, Karnal |  |
| PHT | AX-94833876 | 2D | 30251745 | 8.04E-05 | 9.96 | Delhi, Hisar |  |
| TGW | AX-94384220 | 7D | 188318675 | 1.68E-05 | 10.27 | Delhi, Karnal |  |
| TGW | AX-94684351 | 5B | 440237550 | 1.55E-05 | 11.14 | Delhi, Karnal |  |
| TGW | AX-94955512 | 5A | 564079678 | 0.000288 | 9.63 | Delhi, Karnal |  |
| TGW | AX-95078558 | 7B | 652933607 | 0.000869 | 7.34 | Delhi, Hisar |  |

| Table S5: | | | | | | | | | | | | | | |
| --- | --- | --- | --- | --- | --- | --- | --- | --- | --- | --- | --- | --- | --- | --- |
| Accessions | GY | HI | PHT | GFD | TGW | DM | GFR | | | GNM | | | PTL | BM |
| IC416188 | AX-94518519 (G*****/A)  AX-94991116 (G*/A)  AX-95012948 (T*/G)  AX-94481464 (A*/C) | AX-94401187 (G*/C)  AX-95012948 (T*/G)  AX-94764260 (C*/T)  AX-94916820 (C*/G) |  |  |  |  | AX-94481464 (A*/C)  AX-94433426 (A*/C) | | | AX-95129810 (C*/G)  AX-95012948 (T*/G) | | |  | AX-94433426 (A*/C)  AX-94764260 (C*/T) |
| IC443636 | AX-94518519 (G*/A)  AX-94991116 (G*/A)  AX-95012948 (T*/G)  AX-94481464 (A*/C) |  |  |  |  |  | AX-94481464 (A*/C)  AX-94433426 (A*/C) | | |  | | | AX-95072103 (G*/T)  AX-95189509 (G*/A)  AX-95202740 (C*/T)  AX-94476476 (A*/G) | AX-94860125 (C*/G)  AX-94433426 (A*/C)  AX-94764260 (C*/T) |
| IC335732 | AX-94518519 (G*/A)  AX-94991116 (G*/A)  AX-95012948 (T*/G)  AX-94481464 (A*/C) |  |  |  |  |  | AX-94481464 (A*/C)  AX-94616006 (C*/A) | | |  | | | AX-95090184 (A*/G)  AX-94476476 (A*/G) | AX-94860125 (C*/G) |
| IC536375 | AX-94518519 (G*/A)  AX-94991116 (G*/A)  AX-95012948 (T*/G)  AX-94481464 (A*/C) |  |  |  |  |  |  | | | AX-95129810 (C*/G)  AX-95012948 (T*/G)  AX-94991116 (G*/A) | | |  |  |
| IC128523 | AX-94481464 (A*/C) |  |  |  |  |  | AX-94481464 (A*/C)  AX-94508364 (G*/A)  AX-95104956 (T*/G)  AX-94433426 (A*/C) | | |  | | |  | AX-94433426 (A*/C)  AX-94764260 (C*/T) |
| IC534306 | AX-94518519 (G*/A)  AX-94991116 (G*/A)  AX-95012948 (T*/G)  AX-94481464 (A*/C) |  |  | AX-94853198(A*/C)  AX-94538863(C*/T)  AX-94433426 (A*/C) |  |  |  | | |  | | |  |  |
| IC252655 | AX-94518519 (G*/A)  AX-95012948 (T*/G)  AX-94481464 (A*/C)  AX-94991116 (G*/A) |  |  | AX-94833876(G*/A)  AX-94853198(A*/C)  AX-94538863(C*/T)  AX-94433426 (A*/C)  AX-95078558(C*/T) |  |  |  | | |  | | |  |  |
| DBW93 | AX-94481464 (A*/C) |  |  |  |  |  | AX-94481464 (A*/C)  AX-94508364 (G*/A)  AX-95104956 (T*/G)  AX-94434258 (T*/C)  AX-94616006 (C*/A)  AX-94433426 (A*/C) | | |  | | | AX-95072103 (G*/T)  AX-95090184 (A*/G)  AX-94675758 (G*/A)  AX-95189509 (G*/A)  AX-94449793 (G*/A)  AX-95210974 (T*/G)  AX-94997935 (G*/A)  AX-95095444 (G*/T)  AX-95133008 (G*/A)  AX-94476476 (A*/G) | AX-94433426 (A*/C)  AX-94764260 (C*/T) |
| RAJ4083 | AX-94518519 (G*/A)  AX-95012948 (T*/G)  AX-94481464 (A*/C)  AX-94991116 (G*/A) |  |  |  | AX-94684351 (C*/T)  AX-95078558 (C*/T)  AX-94691823 (A*/C)  AX-94433426 (A*/C) |  | | AX-94481464 (A*/C)  AX-94433426 (A*/C) | | |  | | AX-95090184 (A*/G)  AX-94675758 (G*/A)  AX-95189509 (G*/A)  AX-94842402 (A*/C)  AX-94449793 (G*/A)  AX-95210974 (T*/G)  AX-95202740 (C*/T)  AX-94997935 (G*/A)  AX-95095444 (G*/T)  AX-95133008 (G*/A)  AX-94476476 (A*/G) | AX-94860125 (C*/G)  AX-94433426 (A*/G)  AX-94764260 (C*/T) |
| HGP1-306 |  | AX-94401187 (G*/C)  AX-95012948 (T*/G)  AX-94916820 (C*/T)  AX-94626763 (T*/G)  AX-94764260 (C*/T) |  |  |  |  | |  | | |  | |  |  |
| HD2932 | AX-94518519 (G*/A)  AX-95012948 (T*/G)  AX-94481464 (A*/C)  AX-94991116 (G*/A) |  |  |  |  |  | | AX-94481464 (A*/C)  AX-94508364 (G*/A)  AX-95104956 (T*/G)  AX-94434258 (T*/C) | | |  | | AX-95072103 (G*/T)  AX-95090184 (A*/G)  AX-95189509 (G*/A)  AX-94449793 (G*/A)  AX-95210974 (T*/G)  AX-94997935 (G*/A)  AX-95095444 (G*/T)  AX-95133008 (G*/A)  AX-94476476 (A*/G)  AX-94383522 (C*/T)  AX-95207464 (G*/C)  AX-94842402 (A*/C) | AX-94860125 (C*/G)  AX-94764260 (C*/T) |
| HD2888 | AX-94518519 (G*/A)  AX-95012948 (T*/G)  AX-94481464 (A*/C)  AX-94991116 (G*/A) |  |  |  | AX-94433426(A*/C) |  | | | AX-94481464 (A*/C)  AX-94508364 (G*/A)  AX-94434258 (T*/C)  AX-94616006 (C*/A)  AX-94433426 (A*/C) | | |  | AX-95090184 (A*/G)  AX-94675758 (G*/A)  AX-95189509 (G*/A)  AX-94449793 (G*/A)  AX-95210974 (T*/G)  AX-94476476 (A*/G)  AX-94383522 (C*/T) | AX-94860125 (C*/G)  AX-94433426 (A*/C) |
| K8027 | AX-94518519 (G*/A)  AX-95012948 (T*/G)  AX-94481464 (A*/C)  AX-94991116 (G*/A) |  |  |  |  |  | | | AX-94481464 (A*/C)  AX-94434258 (T*/C)  AX-94616006 (C*/A)  AX-94433426 (A*/C) | | |  | AX-95072103(G*/T)  AX-94675758 (G*/A)  AX-94842402 (A*/C)  AX-95189509 (G*/A)  AX-94449793 (G*/A)  AX-95202740 (C*/T)  AX-95210974 (T*/G)  AX-94383522 (C*/T)  AX-95207464 (G*/C) |  |
| HD3118 | AX-94518519 (G*/A)  AX-95012948 (T*/G)  AX-94481464 (A*/C)  AX-94991116 (G*/A) | AX-94401187 (G*/C)  AX-95012948 (T*/G)  AX-94764260 (C*/T)  AX-94916820 (C*/G)  AX-95078558 (C*/T) |  |  |  |  | | | AX-94481464 (A*/C)  AX-94508364 (G*/A)  AX-95104956 (T*/G)  AX-94434258 (T*/C)  AX-94616006 (C*/A)  AX-94433426 (A*/C) | | |  | AX-95072103 (G*/T)  AX-95090184 (A*/G)  AX-94675758 (G*/A)  AX-94842402 (A*/C)  AX-94449793 (G*/A)  AX-95202740 (C*/T)  AX-94476476 (A*/G) |  |
| DBW107 |  |  |  |  | AX-94684351 (C*/T)  AX-95078558 (C*/T)  AX-94691823 (A*/C)  AX-94433426 (A*/C)  AX-94918415 (A*/G) |  | | | AX-94508364 (G*/A)  AX-95104956 (T*/G)  AX-94434258 (T*/C)  AX-94433426 (A*/C) | | |  | AX-95072103 (G*/T)  AX-95090184 (A*/G)  AX-94675758 (G*/A)  AX-94842402 (A*/C)  AX-94449793 (G*/A)  AX-95202740 (C*/T)  AX-94476476 (A*/G) |  |
| WH1021 |  |  |  |  |  |  | | | AX-94434258 (T*/C)  AX-94433426 (A*/C) | | |  | AX-95072103 (G*/T)  AX-95090184 (A*/G)  AX-94675758 (G*/A)  AX-94842402 (A*/C)  AX-95189509 (G*/A)  AX-94449793 (G*/A)  AX-95210974 (T*/G)  AX-94997935 (G*/A)  AX-95095444 (G*/T)  AX-95133008 (G*/A)  AX-94383522 (C*/T)  AX-95207464 (G*/C)  AX-94476476 (A*/G) |  |
| PBW644 | AX-94518519 (G*/A)  AX-95012948 (T*/G)  AX-94481464 (A*/C)  AX-94991116 (G*/A) |  |  |  |  |  | | | AX-94481464 (A*/C)  AX-95104956 (T*/G)  AX-94434258 (T*/C)  AX-94616006 (C*/A)  AX-94433426 (A*/C) | | |  | AX-95072103 (G*/T)  AX-95090184 (A*/G)  AX-94675758 (G*/A)  AX-94842402 (A*/C)  AX-95189509 (G*/A)  AX-94449793 (G*/A)  AX-95210974 (T*/G)  AX-94997935 (G*/A)  AX-95095444 (G*/T)  AX-95133008 (G*/A)  AX-94383522 (C*/T) |  |
| WH1142 | AX-94481464 (A*/C) | AX-94401187 (G*/C)  AX-94764260 (C*/T)  AX-94916820 (C*/G)  AX-94626763 (T*/G)  AX-95078558 (C*/T) |  |  |  |  | | | AX-94481464 (A*/C)  AX-95104956 (T*/G)  AX-94434258 (T*/C)  AX-94616006 (C*/A)  AX-94433426 (A*/C) | | |  | AX-95090184 (A*/G)  AX-94675758 (G*/A)  AX-94842402 (A*/C)  AX-95189509 (G*/A)  AX-95202740 (C*/T)  AX-95210974 (T*/G)  AX-94997935 (G*/A)  AX-95095444 (G*/T)  AX-95133008 (G*/A)  AX-95207464 (G*/C) | AX-94433426 (A*/C)  AX-94764260 (C*/T) |
| C306 | AX-94481464 (A*/C) |  |  |  | AX-94684351 (C*/T)  AX-94691823 (A*/C)  AX-94433426 (A*/C) |  | | |  | | |  | AX-95090184 (A*/G)  AX-94675758 (G*/A)  AX-94842402 (A*/C)  AX-95189509 (G*/A)  AX-94449793 (G*/A)  AX-95210974 (T*/G)  AX-94997935 (G*/A)  AX-95095444 (G*/T)  AX-95133008 (G*/A)  AX-94383522 (C*/T)  AX-95207464 (G*/C) | AX-94433426 (A*/C)  AX-94764260 (C*/T) |
| J31-170 |  | AX-94401187 (G*/C)  AX-94764260 (C*/T)  AX-94916820 (C*/G)  AX-95012948 (T*/G) | AX-94995102 (C*/T)  AX-94518519 (G*/A)  AX-94691823 (A*/C)  AX-94764260 (C*/T)  AX-94833876 (G*/A)  AX-94561691 (A*/C |  | AX-94684351 (C*/T)  AX-94691823 (A*/C)  AX-94433426 (A*/C)  AX-94918415 (A*/G) |  | | |  | | |  |  |  |
| IC336741 |  |  |  | AX-94833876 (G*/A)  AX-95078558(C*/T)  AX-94853198(A*/C)  AX-94538863(C*/T)  AX-94433426 (A*/C) |  |  | | | AX-94481464 (A*/C)  AX-95104956 (T*/G)  AX-94434258 (T*/C)  AX-94433426 (A*/C) | | |  |  | AX-94860125 (C*/G)  AX-94433426 (A*/C)  AX-94764260 (C*/T) |
| EC190878 |  |  |  | AX-95078558(C*/T)  AX-94853198(A*/C)  AX-94433426 (A*/C) |  |  | | | AX-94481464 (A*/C)  AX-95104956 (T*/G)  AX-94433426 (A*/C)  AX-94508364 (G*/A) | | | AX-95129810 (C*/G)  AX-95012948 (T*/G)  AX-94991116 (G*/A)  AX-95207464 (G*/C) |  |  |
| IC539155 |  |  |  | AX-94833876(G*/A)  AX-95078558(C*/T)  AX-94853198(A*/C)  AX-94538863(C*/T)  AX-94433426 (A*/C) |  |  | | |  | | | AX-95012948 (T*/G)  AX-94991116 (G*/C) | AX-95090184 (A*/G)  AX-94675758 (G*/A)  AX-95210974 (T*/G)  AX-94997935 (G*/A)  AX-95095444 (G*/T)  AX-95133008 (G*/A)  AX-94476476 (A*/G) |  |
| IC443694 |  |  |  | AX-95078558(C*/T)  AX-94853198(A*/C)  AX-94538863(C*/T)  AX-94433426 (A*/C) |  |  | | |  | | | AX-95129810 (C*/G)  AX-95012948 (T*/G)  AX-94991116 (G*/C)  AX-95207464 (G*/C) | AX-95072103 (G*/T)  AX-95189509 (G*/A)  AX-94449793 (G*/A)  AX-95210974 (T*/G)  AX-94997935 (G*/A)  AX-95095444 (G*/T)  AX-95133008 (G*/A)  AX-94383522 (C*/T)  AX-95207464 (G*/C)  AX-94476476 (A*/G) |  |
| IC539292 |  |  |  |  |  | AX-94779369 (C*/A)  AX-94853198 (A*/C) | | |  | | | AX-95129810 (C*/G)  AX-95012948 (T*/G)  AX-94991116 (G*/C)  AX-95207464 (G*/C) |  |  |
| 11-F1-3 | AX-94481464(C*/A) |  | AX-94995102 (C*/T)  AX-94518519 (G*/A)  AX-94691823 (A*/C)  AX-94764260 (C*/T) |  | AX-94684351 (C*/T)  AX-95078558 (C*/T)  AX-94691823 (A*/C)  AX-94433426 (A*/C)  AX-94918415 (A*/G) |  | | | AX-94481464 (A*/C) | | | AX-95129810 (C*/G)  AX-95207464 (G*/C) |  |  |
| IC75240 | AX-94481464(C*/A) |  | AX-94833876 (G/A*) |  | AX-94433426 (A*/C) |  | | |  | | |  |  |  |
| IC321906 | AX-94481464(A*/C) |  |  |  |  |  | | | AX-94481464 (A*/C)  AX-94508364 (G*/A)  AX-95104956 (T*/G)  AX-94434258 (T*/C)  AX-94616006 (C*/A)  AX-94433426 (A*/C) | | |  |  | AX-94433426 (A*/C)  AX-94764260 (C*/T) |

| 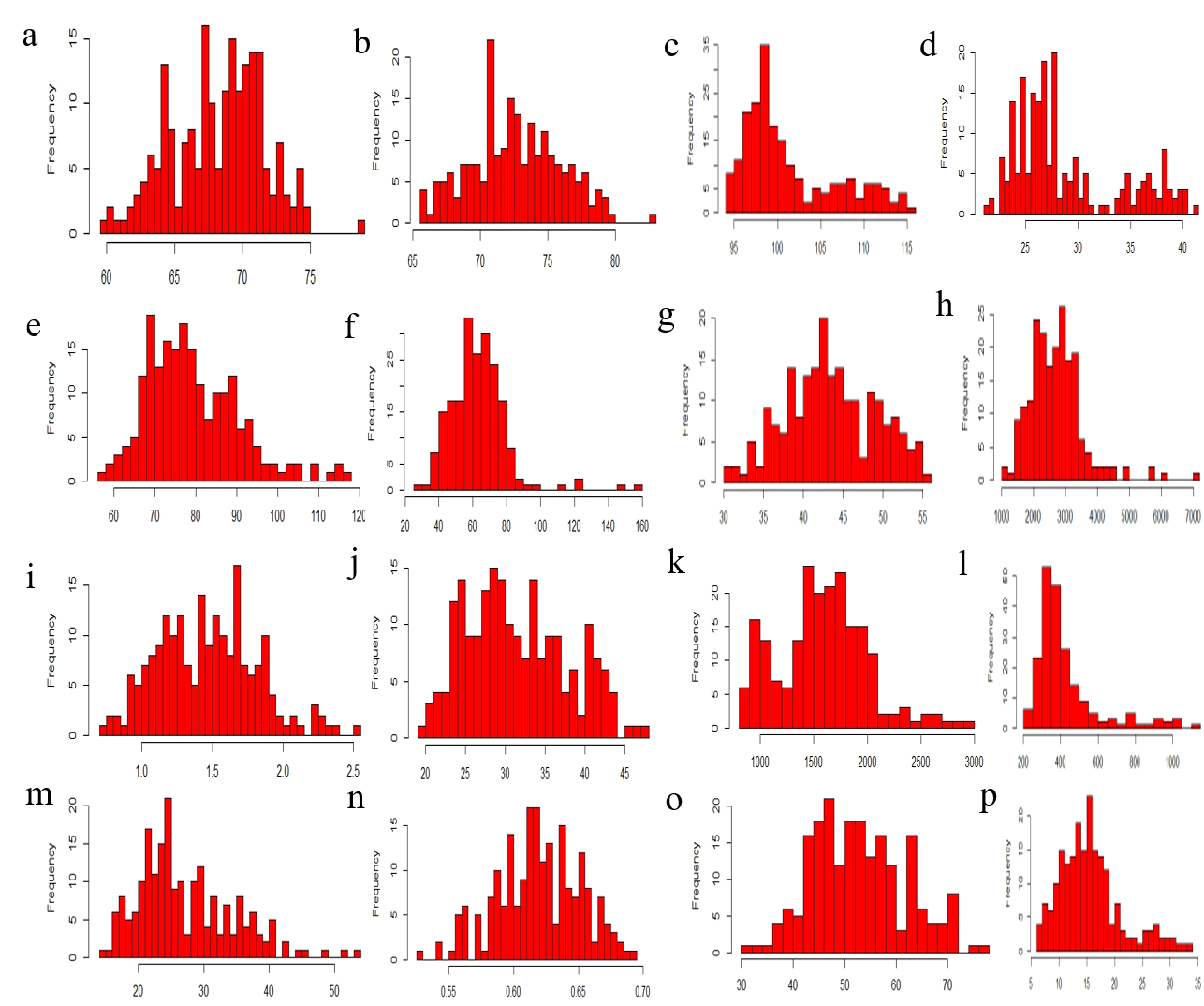 |
| --- |
| Fig. S1: |


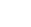


| 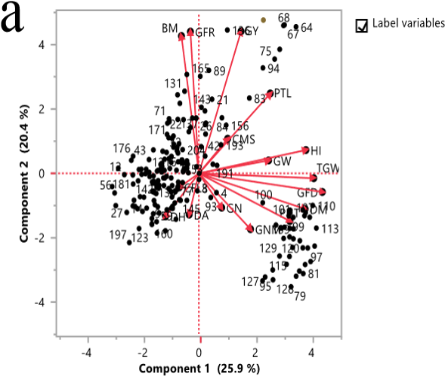 | 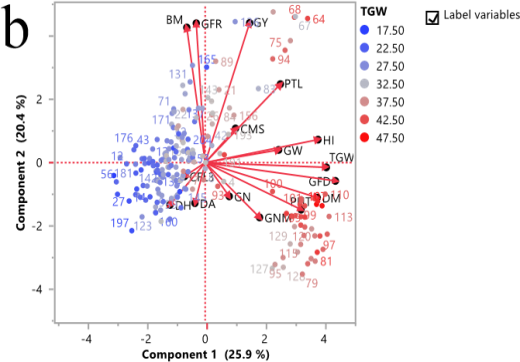 | 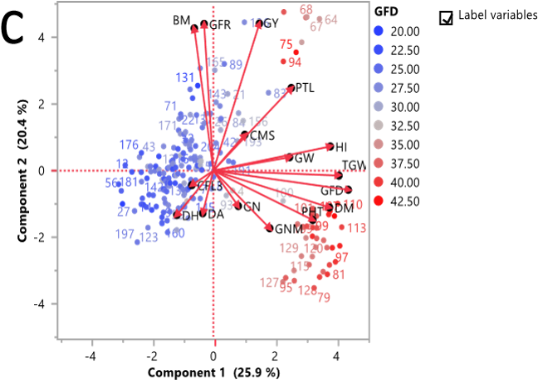 |
| --- | --- | --- |
| Fig. S2: | | |

| 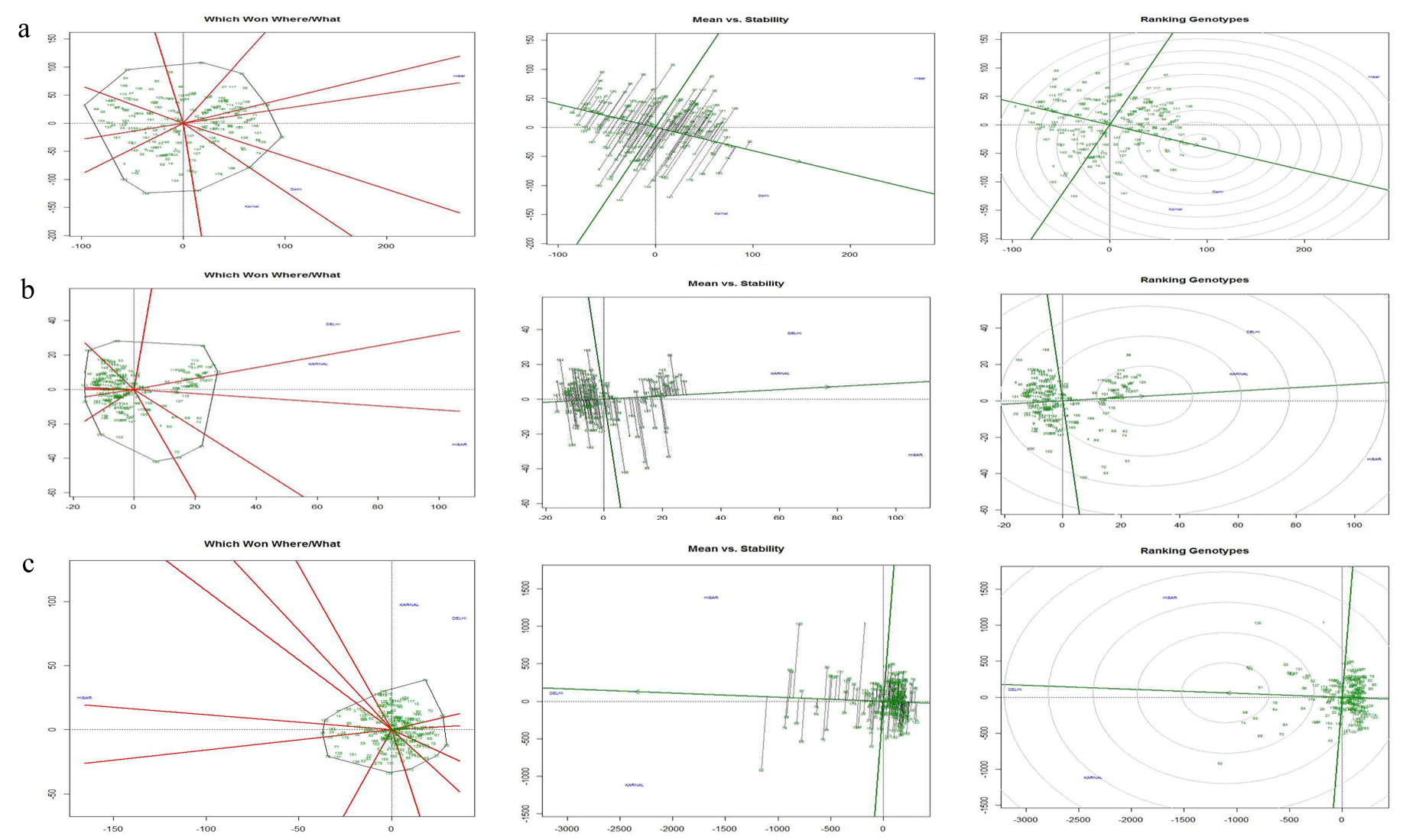 |
| --- |
| Fig. S3: |

| 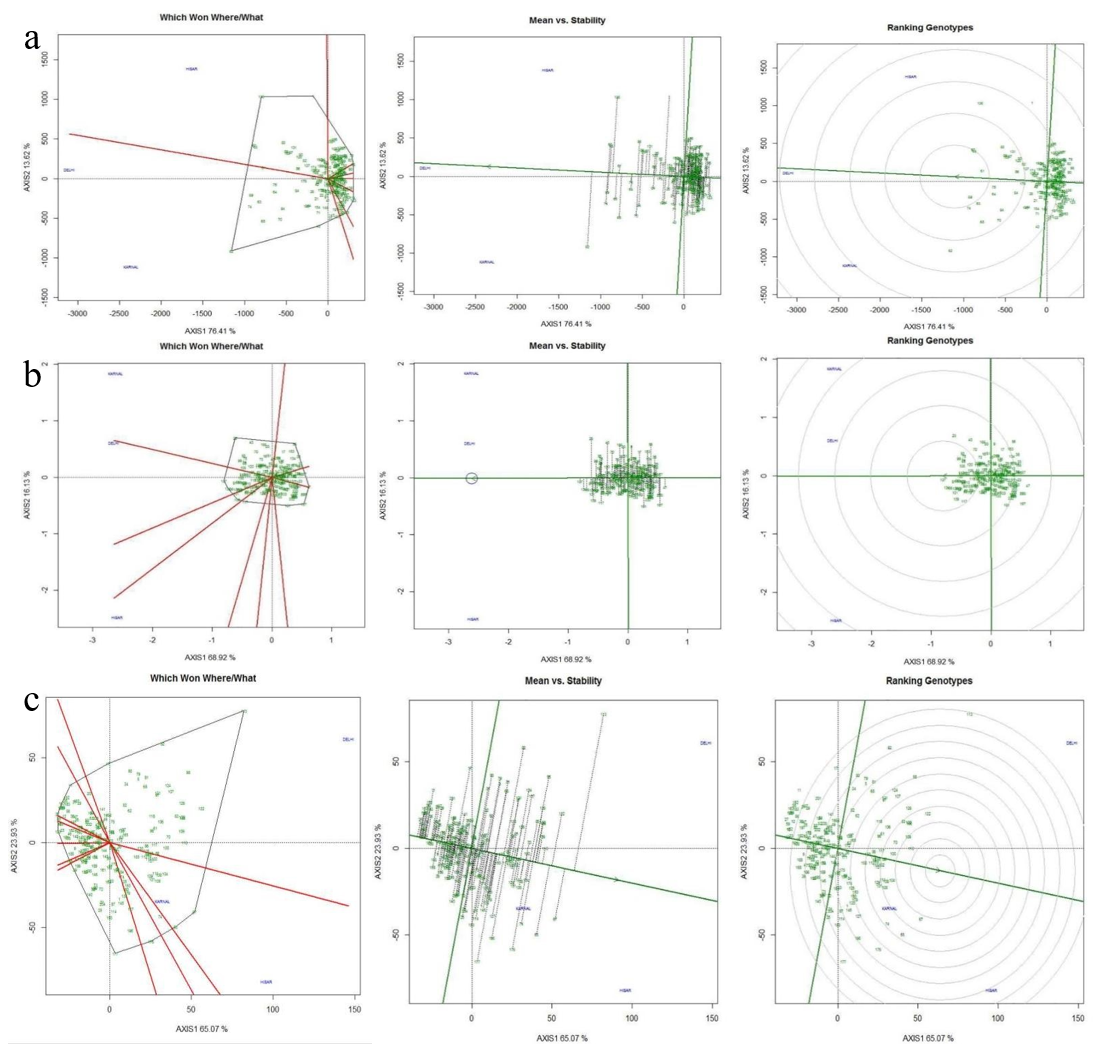 |
| --- |
| Fig. S4: |

| 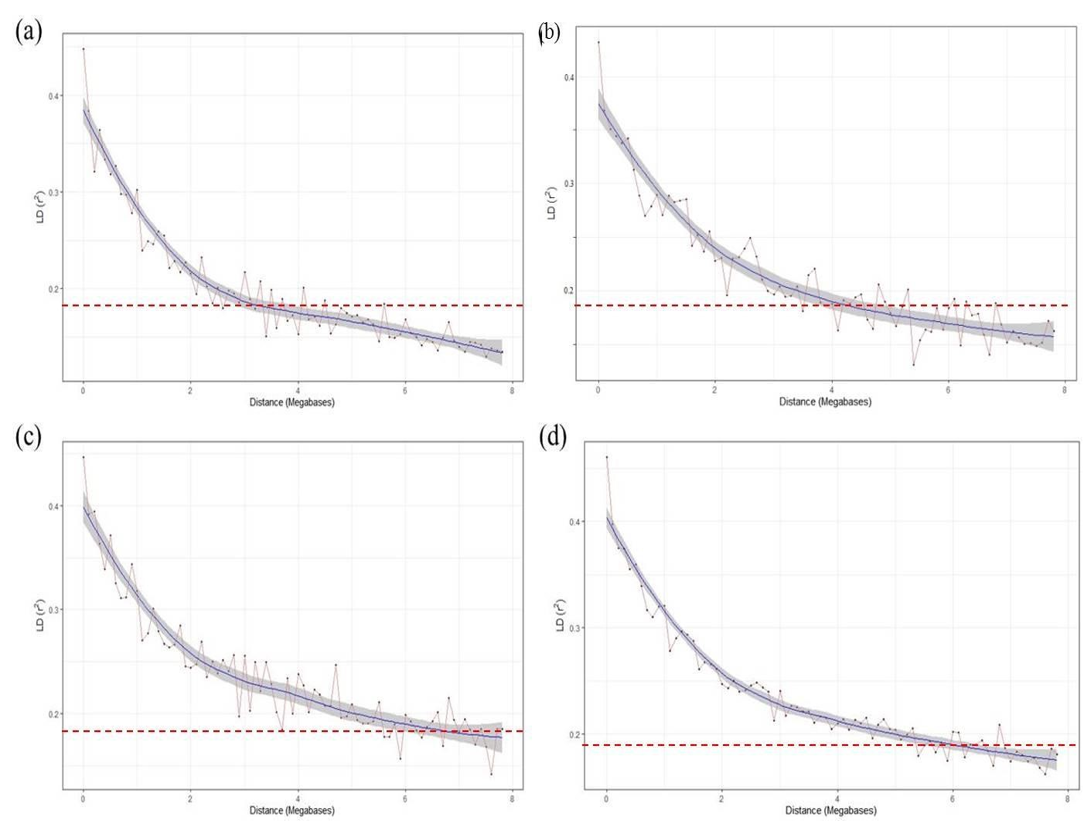 |
| --- |
| Fig. S5: |

| 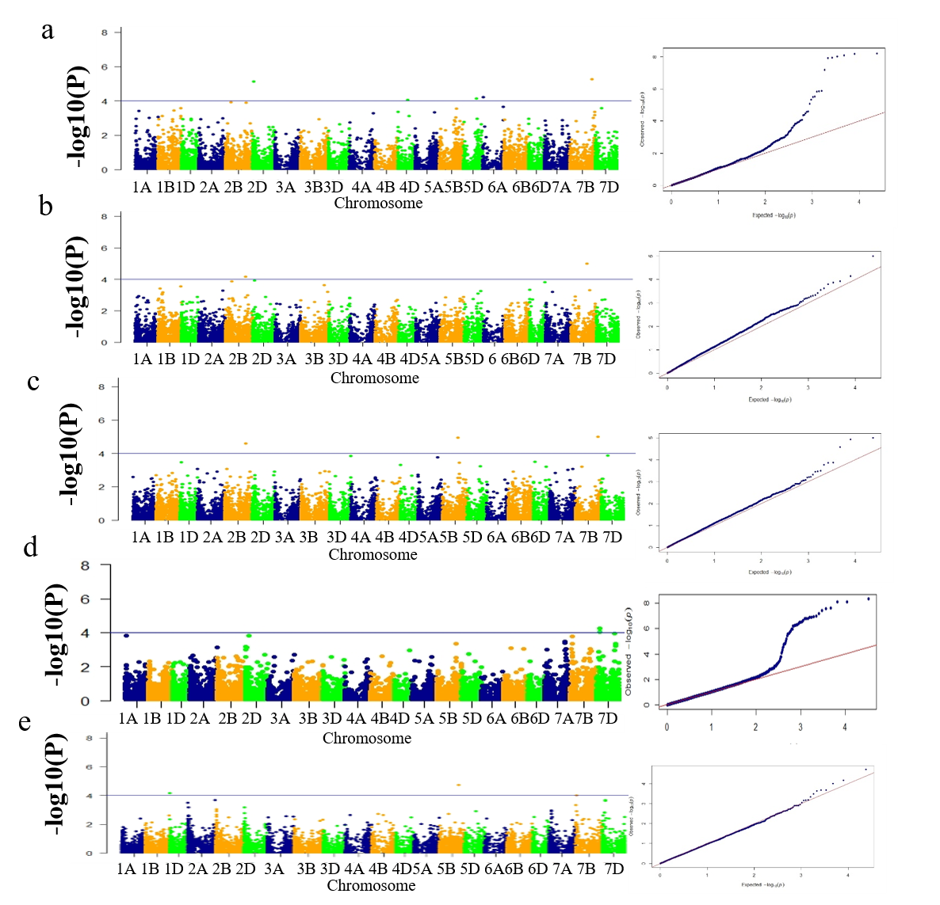 |
| --- |
| Fig. S6: |

| 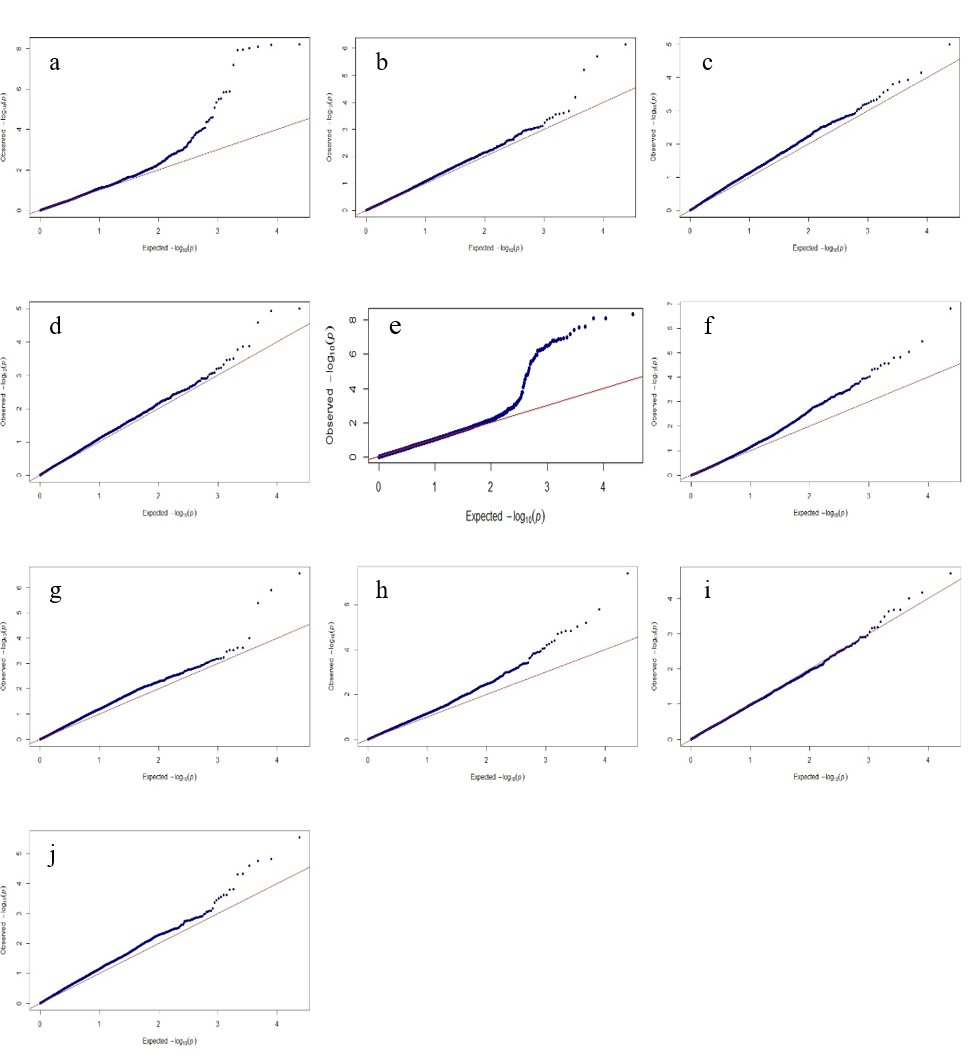 |
| --- |
| Fig. S7: |
